# Internationalization of Read-Across as a Validated New Approach Method (NAM) for Regulatory Toxicology

**DOI:** 10.14573/altex.1912181

**Published:** 2020-04-30

**Authors:** Costanza Rovida, Tara Barton-Maclaren, Emilio Benfenati, Francesca Caloni, P. Charukeshi Chandrasekera, Christophe Chesné, Mark T. D. Cronin, Joop De Knecht, Daniel R. Dietrich, Sylvia E. Escher, Suzanne Fitzpatrick, Brenna Flannery, Matthias Herzler, Susanne Hougaard Bennekou, Bruno Hubesch, Hennicke Kamp, Jaffar Kisitu, Nicole Kleinstreuer, Simona Kovarich, Marcel Leist, Alexandra Maertens, Kerry Nugent, Giorgia Pallocca, Manuel Pastor, Grace Patlewicz, Manuela Pavan, Octavio Presgrave, Lena Smirnova, Michael Schwarz, Takashi Yamada, Thomas Hartung

**Affiliations:** 1 Center for Alternatives to Animal Testing, CAAT-Europe, University of Konstanz, Konstanz, Germany; 2 Existing Substances Risk Assessment Bureau, Health Canada, Ottawa, Canada; 3 Laboratory of Environmental Chemistry and Toxicology, Department of Environmental Health Sciences, Istituto di Ricerche Farmacologiche Mario Negri IRCCS, Milan, Italy; 4 Università degli Studi di Milano, Department of Veterinary Medicine (DIMEVET) Milan, Milan, Italy; 5 Canadian Centre for Alternatives to Animal Methods, University of Windsor, Ontario, Canada; 6 Biopredic International, Saint Grégoire, France; 7 Liverpool John Moores University, School of Pharmacy and Biomolecular Sciences, Liverpool, UK; 8 Centre for Safety of Substances and Products, National Institute for Public Health and the Environment (RIVM), Bilthoven, The Netherlands; 9 Human and Environmental Toxicology, University of Konstanz, Konstanz, Germany; 10 Fraunhofer Institute for Toxicology and Experimental Medicine (ITEM), Hannover, Germany; 11 US Food and Drug Administration, Center for Food Safety and Applied Nutrition, MD, USA; 12 German Federal Institute for Risk Assessment (BfR), Berlin, Germany; 13 Danish Environmental Protection Agency, Copenhagen, Denmark / Danish Technical University, FOOD, Lyngby, Denmark; 14 European Chemical Industry Council (Cefic), Brussels, Belgium; 15 Experimental Toxicology and Ecology, BASF SE, Ludwigshafen, Germany; 16 In vitro Toxicology and Biomedicine, Dept inaugurated by the Doerenkamp-Zbinden Foundation, University of Konstanz, Konstanz, Germany; 17 NTP Interagency Center for the Evaluation of Alternative Toxicological Methods (NICEATM), National Institute of Environmental Health Sciences, National Institutes of Health, Research Triangle Park, NC, United States; 18 S-IN Soluzioni Informatiche S.r.l., Vicenza, Italy; 19 Center for Alternatives to Animal Testing (CAAT), Johns Hopkins University, Baltimore, MD, USA; 20 Australian Government Department of Health, Canberra, Australia; 21 Research Programme on Biomedical Informatics (GRIB), Institut Hospital del Mar d’Investigacions Mèdiques (IMIM), Dept. of Experimental and Health Sciences, Universitat Pompeu Fabra, Barcelona, Spain; 22 Center for Computational Toxicology & Exposure (CCTE), U.S. Environmental Protection Agency, Research Triangle Park, NC, USA; 23 Innovatune S.r.l., Padova, Italy; 24 Departamento de Farmacologia e Toxicologia, Instituto Nacional de Controle da Qualidade em Saúde, Fundação Oswaldo Cruz (Fiocruz), Rio de Janeiro, Brazil; 25 University of Tübingen, Tübingen, Germany; 26 National Institute of Health Sciences, Kanagawa, Japan

## Abstract

Read-across (RAx) translates available information from well-characterized chemicals to a substance for which there is a toxicological data gap. The OECD is working on case studies to probe general applicability of RAx, and several regulations (e.g., EU-REACH) already allow this procedure to be used to waive new *in vivo* tests. The decision to prepare a review on the state of the art of RAx as a tool for risk assessment for regulatory purposes was taken during a workshop with international experts in Ranco, Italy in July 2018. Three major issues were identified that need optimization to allow a higher regulatory acceptance rate of the RAx procedure: (i) the definition of similarity of source and target, (ii) the translation of biological/toxicological activity of source to target in the RAx procedure, and (iii) how to deal with issues of ADME that may differ between source and target. The use of new approach methodologies (NAM) was discussed as one of the most important innovations to improve the acceptability of RAx. At present, NAM data may be used to confirm chemical and toxicological similarity. In the future, the use of NAM may be broadened to fully characterize the hazard and toxicokinetic properties of RAx compounds. Concerning available guidance, documents on Good Read-Across Practice (GRAP) and on best practices to perform and evaluate the RAx process were identified. Here, in particular, the RAx guidance, being worked out by the European Commission’s H2020 project EU-ToxRisk together with many external partners with regulatory experience, is given.

## 1 Introduction

### 1.1 Background

In 2006, the REACH Regulation (Registration, Evaluation, Authorisation and Restriction of Chemicals, Regulation EC 1907/2006) was adopted in the EU, starting a new era of chemical assessment and asking for an unprecedented level of effort in collecting toxicological data on chemicals that were already on the market (Hartung, 2010a). Under REACH, importers and manufacturers of chemical substances are obliged to submit a registration dossier to the European Chemicals Agency (ECHA) containing an extensive list of data on the intrinsic properties of a substance, ranging from chemical characterization and physicochemical properties to toxicological and ecotoxicological data, with increasing demands depending on the tonnage band of the quantity of the substance placed on the market. In addition, registrants should collect information on use and exposure to perform a chemical safety assessment (CSA) based on the toxicity profile of the substance. The minimum data requirements are described in Annexes VI-X of the regulation. For toxicological testing, the standard requirements ask for *in vivo* or *in vitro* tests according to Regulation EC 440/2008, though tests performed according to OECD (Organisation for Economic Co-operation and Development) guidelines are generally accepted. Rovida and Hartung (2009) predicted that full compliance with the REACH Regulation would have required an enormous number of animals. This did not happen because fewer chemicals than expected were registered and because REACH permits waiving of the standard studies by applying a suitable adaptation to the standard required endpoint information. This opportunity is detailed in Annex XI of the REACH regulation, which also defines grouping of substances and the read-across (RAx) approach as: “*Substances whose physicochemical, toxicological and ecotoxicological properties are likely to be similar or follow a regular pattern as a result of structural similarity may be considered as a group or ‘category’ of substances. Application of the group concept requires that physicochemical properties, human health effects, and environmental effects or environmental fate may be predicted from data for reference substance(s) within the group by interpolation to other substances in the group (read-across approach). This avoids the need to test every substance for every endpoint*.”

Generally speaking, RAx is a technique used to predict endpoint information for one chemical by using data from the same endpoint from (an)other chemical(s) that is considered to be similar in some way. If data are read across from one or only a few source substances, this is often called an “analogue approach”. In contrast, grouping refers to the definition of a set of substances combined in a category, where their known toxicological properties follow a specific trend that can be used to infer the properties of the chemicals belonging to it. The precise border of distinction is not defined, because one-to-one comparison is often supported by evidence gained with other, similar chemicals. For this reason, RAx, as used in this report, represents any approach where a data gap is filled using existing information obtained for other substances (Patlewicz et al., 2014).

REACH Annex XI describes also other approaches to waive tests, including quantitative structure activity relationships (QSARs), weight-of-evidence (WoE), and *in vitro* testing. Among the non-testing approaches, no distinct edge between RAx, WoE or QSAR can be defined, and thus these three techniques may be combined.

According to the latest ECHA report (ECHA, 2017a) on the general use of alternative methods for REACH purposes, RAx has been extensively used in REACH registration dossiers, in particular for human health data endpoints. Indeed, alternative strategies have been used in about 34% of the dossiers for lower tier toxicological endpoints, e.g., local and acute endpoints for substances up to 100 t/y, whereas alternative strategies were employed in nearly 84% of the dossiers for substances above 100 t/y in the assessment of higher tier endpoints, e.g., repeated dose toxicity, and reproductive and developmental toxicity. The evaluation of these dossiers by ECHA found overall poor quality of the information provided, leading to low acceptance rates, as outlined by Ball et al. (2016) and ECHA. This was corroborated by a report and workshop organized by the German Federal Institute for Risk Assessment (BfR, 2018a,b). In many cases, RAx justifications by the registrants lacked important information, specifically with regard to explaining the influence of structural differences between source(s) and target(s), and justification of the inclusion/exclusion of group members. However, when performed correctly, RAx is probably the most direct approach for adapting standard information requirements.

Under Title VI, Chapter 1 (Dossier Evaluation) of the REACH Regulation, ECHA must evaluate the submitted dossiers for compliance with regulatory requirements. To that end, ECHA has implemented a system to manually check all dossiers containing waiving of standard information, as is the case when RAx is included in a REACH registration dossier. In this context, rejecting RAx due to inadequate justification has been shown to be one of the main reasons for requesting further (i.e., always *in vivo*) data from the registrants. Notably, a RAx justification will be rejected when ECHA considers the RAx hypothesis by the registrants implausible but also if the documentation provided is inadequate to allow a reliable assessment of its soundness. Given ECHA’s announcement to substantially increase their dossier evaluation activities, starting already in 2019^1^, it is clear that adequately documenting valid RAx hypotheses in a scientifically sound way is a key issue when trying to avoid unnecessary animal testing under REACH.

Regarding RAx, ECHA issued a dedicated document describing the Read-Across Assessment Framework (RAAF) in 2015, which was updated in 2017 to include multi-constituent substances and substances of unknown or variable composition, complex reaction products and biological materials (UVCB) (ECHA, 2017b,c). Originally, these publications were intended only to define the rules applied by ECHA when assessing the RAx strategies. Like any framework, the RAAF document provides high-level information on the scope and the key elements a valid RAx justification should contain but provides no detailed guidance on how a RAx justification should be prepared. It also lacks practical examples of accepted or rejected RAx justifications. However, the RAAF document is very useful, as it describes the basic principles of RAx, which should be supported by both strong chemical and biological similarity, and a scoring system for rating the acceptability of the RAx strategy. Notably, the RAAF was published late in the REACH process, i.e., when most of the REACH registration dossiers were already submitted or at an advanced stage of preparation. Being published in 2015, the RAAF was available only to REACH phase-in registrants submitting dossiers for the final deadline, May 30, 2018, which included all substances marketed in the tonnage band above 1 t/y.

As far back as 2012, the European Centre for Ecotoxicology and Toxicology of Chemicals (ECETOC) published a technical report on the category approach, RAx and QSAR (ECETOC, 2012) that represents an introduction to the topic but provides only minor guidance on how to prepare and submit a RAx justification document to the regulatory authorities. Many of the recommendations proposed in the ECETOC document were later incorporated in an OECD guidance document for grouping that was published in 2014 (OECD, 2014) and updated in 2017 (OECD, 2017a). The latter describes the difference(s) between the analogue approach, where the comparison between source and target is mostly one-to-one and there is no difference in activity, and the category approach, where the toxicological assessment is performed on a group of chemicals for which the specific endpoint has a regular pattern or trend within the category. The innovative aspect was to provide some more guidance on how to go about crafting the RAx justification and start the discussion about where *in vitro* testing and adverse outcome pathways (AOPs) could play a role.

**Fig. 1:**
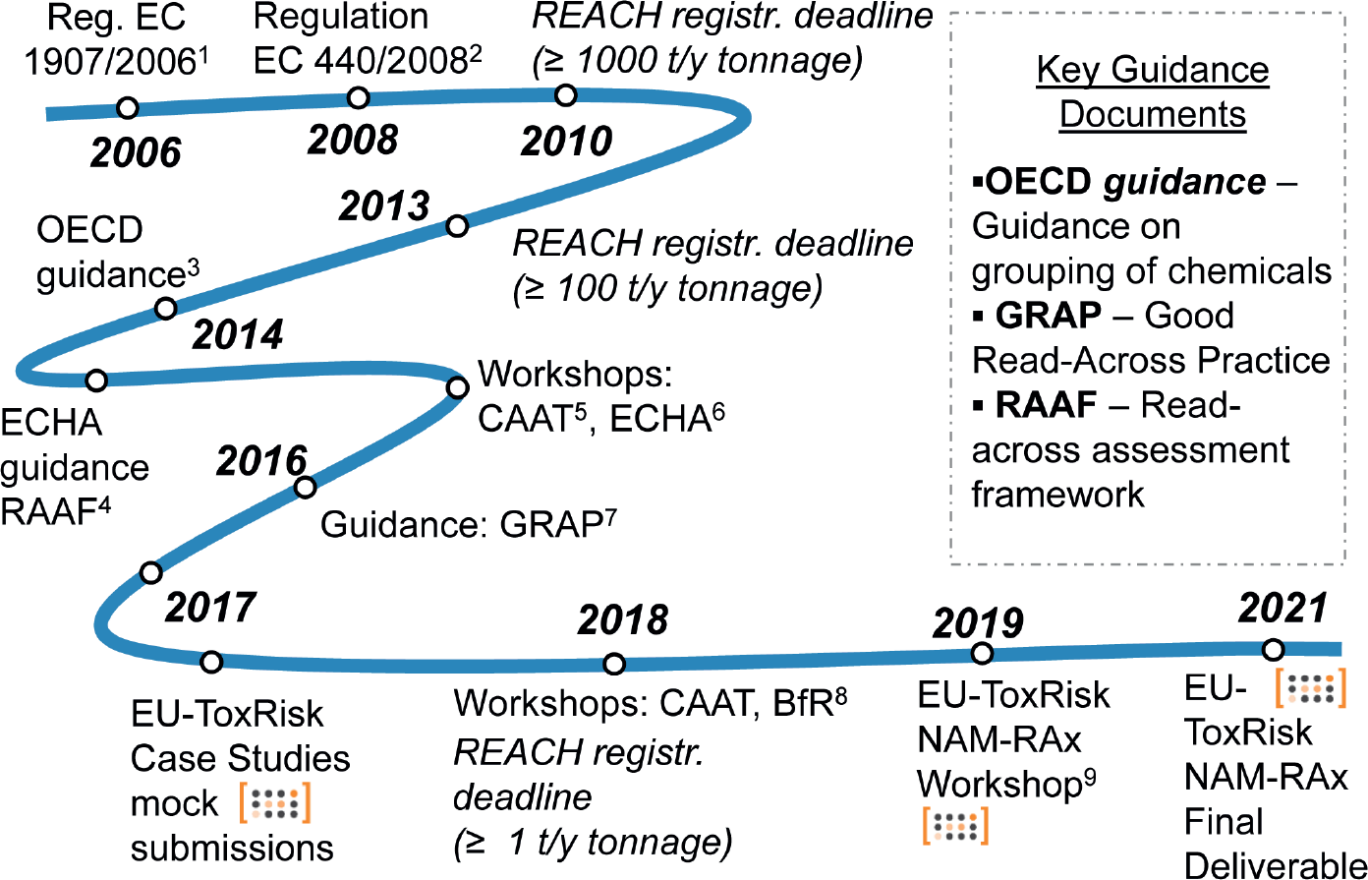
Timeline of the development of RAx application for risk assessment Schematic representation illustrating the key dates and documents for the development of the RAx application for regulatory purposes in Europe and within the EU-ToxRisk Project. Reference documents as mentioned in the text: ^1^ http://data.europa.eu/eli/reg/2006/1907/oj; ^2^ http://data.europa.eu/eli/reg/2008/440/oj; ^3^ https://bit.ly/2IqOBJB; ^4^ https://echa.europa.eu/documents/10162/13628/raaf_en.pdf; ^5^ Maertens et al., 2016; ^6^ ECHA, 2017b; ^7^ Ball et al., 2016; ^8^ https://bit.ly/3jVbYJ9; ^9^ https://bit.ly/3dl4Ing. The EU-ToxRisk final deliverable will represent an advisory document, complimentary to the already published ECHA reporting template for grouping and read-across (ECHA RAAF), facing the issue of regulatory acceptance from the point of view of the registrants of NAM-supported read-across dossiers.

Acknowledging the needs for further guidance and agreement on how to build and apply a good RAx strategy, some initiatives were undertaken in the EU and in the US (Fig. 1). In 2016, CAAT-Europe, Cefic-LRI and EU-ToxRisk hosted a workshop (Maertens et al., 2016) to initiate a discussion on Good Read Across Practice (GRAP). This was followed by a similar initiative in the US^2^ with the collaboration of the US Food and Drug Administration’s Center for Food Safety and Applied Nutrition (FDA-CFSAN) accompanied by a satellite meeting at the Annual Meeting of the Society of Toxicology (SOT) in 2018 relating to the specific topic of regulatory acceptance of RAx (Chesnut et al., 2018). The intent was to summarize the state-of-the-art and provide examples and strategies for how to provide RAx justification based on the experience of industry registrants at the time.

A complementary document (Zhu et al., 2016) described the possible contribution of biological information to structure-based RAx. These concepts were also discussed at a workshop organized by ECHA in 2016 (ECHA, 2016, 2017d), arriving at the conclusion that new *in vitro* tests can be applied to justify RAx approaches. For the first time, the term “new approach methodologies” (NAMs) was introduced and taken “*in a broad context to include* in silico *approaches*, in chemico *and* in vitro *assays, as well as the inclusion of information from the exposure of chemicals in the context of hazard assessment*”. NAMs also embrace other information on toxicodynamics (e.g., high-throughput screening and high content-methods) and toxicokinetics with the aim of improving the understanding of toxic effects (van Vliet et al., 2014).

Currently, the EU-ToxRisk project, which is an Integrated European “Flagship” Programme driving mechanism-based toxicity testing and risk assessment for the 21^st^ century (Daneshian et al., 2016), aims to move away from observational toxicology based on animal models and progress towards a toxicological assessment based on *in vitro* test responses using human cells together with a better mechanistic understanding of chemical adverse effects. The research is focused on two *in vivo* endpoints: repeated dose systemic toxicity (e.g., Hiemstra et al., 2019; Albrecht et al., 2019; Delp et al., 2019,) and developmental and reproductive toxicity (e.g., Rempel et al., 2015; Nyffeler et al., 2017; Delp et al., 2018). One of the first applications of the in EU-ToxRisk *in vitro* and *in silico* test batteries is applying the RAx hypothesis (Graepel et al., 2019; Escher et al., 2019). With this goal, the project has developed *ad hoc* case studies to be reviewed by regulators from ECHA and EU Member States with the aim of further improving the understanding of what constitutes an acceptable RAx justification and how this justification can be supported and improved using NAM data. The integrated results were submitted to regulators from ECHA and EU Member State competent authorities as mock dossiers and discussed in a workshop with stakeholders from academia, industry and authorities in May 2019 (manuscript in preparation).

The ongoing general discussion on RAx has confirmed the complexity of the approach and the need to establish suitable rules for its objective application, leading to a broader regulatory acceptance of RAx for the risk assessment of chemicals. With this goal, a group of stakeholders, coming from many different areas, expertise and organizations, including regulatory, validation and government bodies, was convened in Ranco (Varese, Italy) on July 16–18, 2018. During that workshop, sponsored and co-organized by CAAT-Europe, the EU-ToxRisk project and the Doerenkamp-Zbinden Foundation (DZF), it was decided to prepare an extensive document to present the state-of-the-art of RAx for regulatory purposes with the addition of further comments to foster its applicability for regulatory purposes. The present report, which benefits also from the contribution of experts who did not participate in that meeting, has the ambitious goal to describe the basis for immediate improvement of RAx acceptance as well as to define the strategy for future development in a contribution that may serve as an educational text both for those who are facing the argument for the first time and those who want to deepen it. The concepts and ideas presented here come from the individual participants and do not necessarily reflect the views and opinions of the organizations they represent. The debates were based on scientific discussions among the participants, without a necessarily unanimous final agreement on all issues.

### 1.2 The international dimension of RAx

**Fig. 2:**
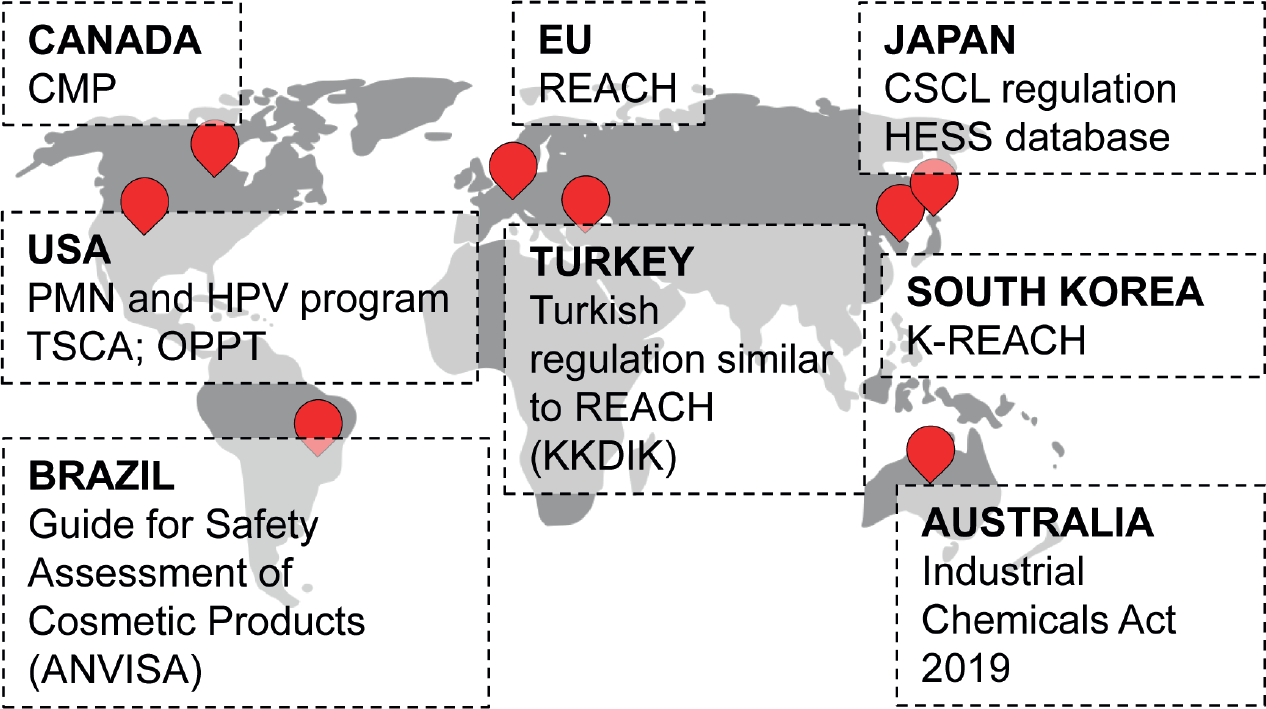
Overview of the current legislations covering RAx regulatory applications worldwide ANVISA, Agência Nacional de Vigilância Sanitária (Brazilian National Health Surveillance Agency); CMP, Chemicals Management Plan; CSCL, Chemical Substances of Control Law; HESS, Hazard Evaluation Support System; HPV, High Production Volume; OPPT, Office of Pollution Prevention and Toxics; PMN, Pre-Manufacture Notice; REACH, Registration, Evaluation, Authorisation and Restriction of Chemicals; TSCA, Toxic Substances Control Act. This list is not intended to be complete.

The EU was the first group of countries to accept RAx as a tool to address information requirements *in lieu* of standard testing, but for proper implementation of the new approach, greater international acceptance is necessary. RAx, and in more general terms *in silico* methods, are now included and promoted in many regulations across countries and sectors. As an example, the situation in some countries is considered here (Fig. 2), without the claim of providing an extensive analysis.

In the US, there is minimal application of RAx for official regulatory dossiers, and there are no formal protocols, procedures or guidance documents on its use. To date, the most widely known use cases in the US are those within the US Environmental Protection Agency (EPA) that already have been captured in the current OECD guidance (OECD, 2017), such as experiences under the Pre-Manufacture Notice (PMN) process or High Production Volume (HPV) program (van Leeuwen et al., 2009) as well as the chemical categories used to review new chemicals under the Toxic Substances Control Act (TSCA)^3^. The US EPA’s Office of Pollution Prevention and Toxics (OPPT), whose experience with RAx approaches is relatively mature, has developed the Analogue Identification Methodology^4^ (AIM) and the Chemical Assessment Clustering Engine (ChemACE)^5^, specifically designed to assist, review and prioritize large inventories of chemicals and to facilitate RAx and data gap-filling for untested substances. The activity of OPPT started in 2010, with the publication of the New Chemical Categories document (OPPT, 2010) that was intended to provide an effective tool for EPA reviewers to benefit from the accumulated data and past decisions as precedents. This document collects the different substances grouped by main chemical functional groups that are supposed to result in a specific physicochemical or toxicological property of the molecule. The Interagency Coordinating Committee on the Validation of Alternative Methods (ICCVAM) has shown continued interest in the potential of RAx to waive new tests on vertebrate animals. In 2016, ICCVAM broadcast a dedicated webinar^6^ and established a working group focused on RAx, which was assigned the task of summarizing best practices for the application and implementation of RAx in the different regulatory settings of interest. A recent publication by the ICCVAM RAx working group summarized current needs and opportunities across US agencies, including decision contexts, desired activities, and any available guidance, and provided a list of freely available RAx tools (Patlewicz et al., 2019). This paper also detailed two specific case studies to illustrate how RAx is applied in practice: the EPA evaluation of n-heptanal under the Superfund program and a toxicological risk assessment for a medical device at the US FDA.

In Canada, Health Canada and Environment and Climate Change Canada’s risk assessment programs have continued to increase the use of RAx in risk assessments throughout the evolution of the three phases of the Chemicals Management Plan (CMP). RAx was introduced as a key approach to support risk assessment during Phase 2 of the CMP (Substance Groupings Initiative 2011–2015)^7^, where chemicals began to be assessed as groups based on structural, functional or mechanistic similarities, and is now routinely used in the risk assessment of new and existing substances in Phase 3 (2016–2020). The Government of Canada gained experience in using RAx by contributing to the development of the OECD guidance on the grouping of chemicals and subsequent updates (OECD, 2014, 2017a) and through expert consultation from the CMP Science Committee on best practices for deriving a sufficient rationale to support risk assessment decisions within the context of the CMP^8^. As the CMP in Canada continues to advance and modernize, RAx remains a critical tool contributing to the WoE evaluation^9^ and reducing uncertainty in an assessment, as well as permitting the assessment of substances that would otherwise have insufficient information. It can also provide verification or validation of other data to fill endpoint-specific data gaps and support the identification of needs for further testing. The RAx tools and approaches that have been developed and implemented under the CMP are evolving to facilitate the integration of emerging data sources and NAM data for the formation of chemical categories to support the identification of groups of chemicals as risk assessment priorities in moving forward. Furthermore, there is on-going development of computational approaches for analogue selection for RAx to advance risk assessment through integrated approaches to testing and assessment (IATA) such as that illustrated by the case study for a group of substituted phenols (Webster et al., 2019).

Other countries have adopted legislations very similar to REACH, but not equally advanced in the promotion of alternative methods. For example, in South Korea the structure of K-REACH resembles that of the EU approach but lacks all reference to the possibility to adapt standard information (Ha et al., 2016). This means that the RAx approach is acceptable but not applied. The first deadline for registering substances in the tonnage band above 1000 t/y will be in 2021, and it is now too early for any analysis of this topic.

In Australia, the Industrial Chemicals (Notification and Assessment) Act 1989 will be replaced on 1 July 2020 by the Industrial Chemicals Act 2019^10^. This act contains no details on the toxicological tests that will be in dedicated guidelines still under construction at the time of writing. However, this new law includes the ban on animal tests on cosmetic ingredients.

A candidate country for accession to the EU, Turkey’s program includes the harmonization of its legislation. KKDIK, the Turkish version of REACH, is very similar to the EU’s REACH regulation. However, since KKDIK came into effect only in 2017, with pre-registration and registration deadlines in 2020 and 2023, respectively, it is not yet known to what extent adaptation to standard information requirements will be applied.

The Japanese Hazard Evaluation Support System (HESS) database and platform^11^, which was started in 2012 and has been updated regularly, is promising with respect to RAx application. HESS supports the evaluation of repeated dose toxicity by category approach and is linked to two databases. The first is a toxicity knowledge database, which contains information on repeated-dose toxicity and toxicity mechanisms. The second is a metabolism knowledge database containing rat metabolism maps and information on absorption, distribution, metabolism and excretion (ADME) in rats and humans. HESS allows chemicals to be categorized on the basis of structural, physicochemical and mechanistic similarities and supports the prediction of repeated-dose toxicity for untested chemicals by means of the category approach. Regarding the legislative framework, chemical substances are regulated by the Chemical Substances of Control Law (CSCL) that may accept a RAx approach for hazard assessment on a case-by-case basis upon the formal approval of a scientific committee. However, so far, the application of RAx for regulatory purposes has been limited.

Regarding Brazil, the Guide for Safety Assessment of Cosmetic Products published by the Brazilian National Health Surveillance Agency (ANVISA – Agência Nacional de Vigilância Sanitária) suggests the use of *in silico* models for selection of raw materials in order to avoid the use of unsafe chemicals. It also states that such results must be carefully used and do not fully replace validated *in vitro* or *in vivo* models. For drugs and biological products, impurity mutagenesis must be evaluated by two *in silico* methods (expert rule-based and statistics based). Read-across is not fully acceptable for general toxicity. The Brazilian Center for Validation of Alternative Methods (BraCVAM) encourages the use of the RAx concept, at least at the beginning of a study, as part of the integrated testing strategy (ITS).

### 1.3 Applications

Given its inherent potential for huge economic savings and scientific innovation, industry is, in general, open to the opportunity of RAx, provided that there are clear rules for its applicability and guarantees for regulatory acceptance. Some key examples are provided here.

The petrochemical industry represented by CONCAWE, a division of the European Petroleum Refiners Association, is studying the environmental health and safety of petroleum derivatives. The problem of petroleum products is that they are complex UVCBs with highly variable compositions. Grouping is generally performed through general similarities of analytical characterization and physicochemical characteristics, which are not sufficient in many cases to justify RAx hypotheses. For this reason, CONCAWE has sponsored a new project, called Cat-App^12^, with the final goal of addressing the specific challenge posed by UVCBs such as petroleum chemicals and defining practical strategies for grouping and RAx strategies for a cost-effective solution. The approach is to integrate innovations in (i) *in vitro* testing, (ii) high-throughput genomics, and (iii) integrative data analyses and visualization into a transparent workflow for RAx assessment. This approach offers interesting perspectives; however, it remains to be seen whether the results produced will ultimately affect the formation of more reliable categories that are acceptable from a regulatory point of view.

Another industrial association that is very active in this field is the International Fragrance Association (IFRA). Similar to petroleum products, many fragrances are also UVCBs, often with unclear structures and embracing many different chemical classes. Fragrances are common ingredients of cosmetic products, and their precise toxicological characterization is of the utmost importance. In the case of fragrances, and for the whole cosmetic sector in general, the opportunity of performing risk assessment using the RAx approach is highly appealing (Api et al., 2015). In fact, more than other industry branches, the cosmetics industry needs very accurate animal-free prediction methods for possible adverse health effects in humans due to the prohibition of testing in vertebrates by the EU Cosmetics Regulation (EC) 1223/2009. To demonstrate how this topic is of outstanding importance, the European Union’s Scientific Committee on Consumer Safety (SCCS) has published a call to hire RAx experts, acknowledging that assessment of cosmetic ingredients is increasingly based on this approach.

RAx is also applied in the evaluation of chemical constituents within dietary supplements (Cohen et al., 2018). For example, the US FDA has used computational modelling of the 25 most prevalent substances in Kratom, a material of plant origin, to compare structural similarities with opioid analgesics^13^. They then used this information, together with previously available experimental data, to determine the substances’ biological targets. Also in this area, botanical safety can be evaluated using RAx in combination with the threshold of toxicological concern (TTC), which is the estimated exposure level that is considered of negligible harm (Little et al., 2017).

An emerging field of RAx application is the grouping of nanomaterials. Many different nanomaterials are used in various sectors, ranging from cosmetics to paints to drugs. Specific biological activities of nanomaterials, which may be determined by their shape, size and/or surface properties, might not be fully captured by traditional toxicological methods, and testing all of these materials with *in vivo* methods seems impossible (Hartung, 2010b; Hartung and Sabbioni, 2011). A major challenge of applying grouping and read-across to nanomaterials is the identification of those material properties that are critical for adverse outcomes of nanomaterial exposure (Oomen et al., 2015; Burden et al., 2017). Current concepts for the grouping of nanomaterials for human health risk assessment (e.g., Landsiedel et al., 2017) consider composition, solubility, morphology and/or surface reactivity. These concepts have been developed further within the EU GRACIOUS project^14^. Mech et al. (2018) reviewed the regulatory acceptance of RAx applied to nanomaterials and pointed out that specific guidance for implementing grouping and RAx of nanomaterials still needs to be developed. There is, however, consensus (e.g., OECD, 2016a) that for nanomaterials, particularly nanoforms of the same chemical composition, grouping and RAx can help to reduce testing while still obtaining sufficient information to assess their risks. Under REACH, ECHA has issued a guidance appendix relevant for nanomaterials (ECHA, 2017e). Similarly, this has been highlighted as an option in the EFSA guidance on the application of nanoscience and nanotechnologies in the food and feed chain (EFSA, 2018a). Several case studies on nanomaterial RAx are available for specific endpoints or exposure routes (Arts et al., 2016; Aschberger et al., 2019).

Another sector where the potential of the RAx approach can be important is the assessment of polymers, whose toxicological assessment is seldom performed. In addition to that, the question about the impact of microplastics in the environment is only starting to be considered from a toxicological point of view. The only exceptions are in the application of polymers as food contact materials. In general, risk assessment of food contact materials is performed only on migrating substances.

The field of medical devices is definitely more complex. In the EU, medical devices are in the scope of the new EU Regulation 2017/745, which is still in the transition period until May 26, 2020. When fully in force, this regulation will require far more demanding tests to approve new products, in particular those with chronic exposure. Since it is new, there is time to improve the use of RAx, which is completely absent from the authorization dossiers of existing products. Toxicological assessment of medical devices in the US is very similar to that proposed in the EU, as both legislations ask for ISO test methods. In theory, *in vitro* tests and RAx are not prohibited, but the probability that regulators will request confirmation with *in vivo* tests usually leads applicants to immediately perform *in vivo* methods to avoid the risk of duplicate testing (Rovida, 2010), which increases costs and causes delays to marketing (Kerecman Myers et al., 2017).

Drug safety assessment shares a very similar situation. The European Medicines Agency (EMA), FDA and other governmental institutions with responsibilities in the approval of new drugs declare a willingness to consider any new approach if its scientific validity is demonstrated, but in the end, all substances must undergo extensive *in vivo* tests for acceptance. At present, RAx is nevertheless applied and accepted for the evaluation of drug impurities that cannot be isolated and characterized^15^. In this sense, RAx is formally accepted in the risk assessment of non-tested contaminants (EMA, 2014). Pharmaceutical companies extensively apply *in silico* methods during drug discovery and development to detect drug candidates with potential safety concerns, which are then removed from the development pipeline to save costs and minimize risks.

The EU Plant Protection Products Regulation (Regulation (EU) 1107/2009), while acknowledging the general principle of avoiding unnecessary animal testing, does not make specific reference to RAx. Article 5 of Regulation (EU) 283/2013 (detailing the data requirements for pesticide active substances) states that “*tests on vertebrate animals shall be undertaken only where no other validated methods are available. Alternative methods to be considered shall include* in vitro *methods and* in silico *methods*,” and arguably RAx could be considered an *in silico* method, in particular when supported by *in silico* tools such as the OECD QSAR Toolbox^16^. However, for the assessment of pesticide active ingredients, RAx is practically never used, though it is increasingly applied for pesticide impurities, metabolites and degradation products. RAx is also usually considered in the preliminary assessment during the gathering and organization of existing information (SAPEA, 2018). Only in the case of dietary assessment of pesticide metabolites is RAx formally used and accepted, in particular for the evaluation of genotoxicity (EFSA, 2016).

In contrast, Annex IV of the EU Biocidal Products Regulation (Regulation (EU) 528/2012) allows for the adaptation of standard information requirements in the same way as Annex XI of the REACH Regulation, and therefore analogue/grouping approaches are frequently employed. As an example, RAx was used to assess the similarity of 5-chloro-2-(4-chlorophenoxy)phenol (DCCP), a biocide approved for human hygiene, disinfection, food and animal feeds, to Triclosan, based on similar toxicokinetics measured in hamsters^17^.

Recently, ECHA and EFSA jointly prepared new guidance for the identification of endocrine disruptor properties of pesticides and biocides, in which a RAx opportunity is explicitly mentioned (ECHA/EFSA, 2018).

**Fig. 3:**
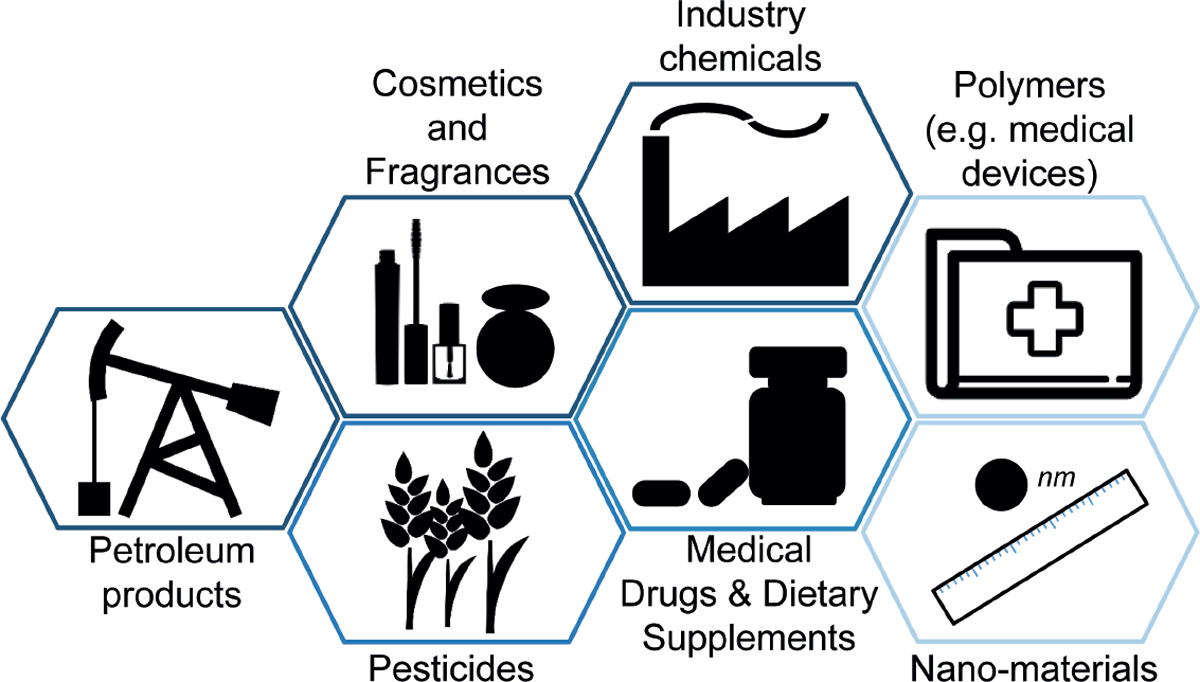
Industry sectors affected by the application of the RAx approach for risk assessment Affected products range from industrial chemicals, cosmetics, medical drugs and pesticides, to petroleum products, nanomaterials, and polymers. Tiles are outlined in shades representing maturity of RAx applications, from darkest (most) to lightest (least).

In conclusion, there are plenty of opportunities for the use of RAx in the different industrial sectors, but they remain substantially underexploited, even though the hope is for a more extensive application in the future (Fig. 3).

## 2 Confidence building

### 2.1 Chemical and biological starting points for similarity assessment

Though this paper is not intended to review all scientific possibilities for RAx justification, it is important to provide the basic principles for the applicability of this approach to facilitate the discussion on validation and acceptance.

A RAx hypothesis should start from a proper definition of the target substance(s) by providing the correct molecular structure and purity. In the case of multi-constituent substances, each component should be defined both with respect to its molecular structure and relative content in the target substance or, in general, the composition should be described as much as possible. Ideally, full spectrometry characterization with mass spectrometry (MS) spectra, nuclear magnetic resonance (NMR) spectra of both hydrogen and carbon, if necessary, an infrared (IR) spectrum, and any other techniques that can be useful for the proper definition of the compound should be provided for each substance. This process should be aimed at the characterization of the whole sample rather than a mere definition of the main component. For this aim, the ECHA guidance for substance identification can be very helpful (ECHA, 2017f).

Once the characterization of the target substance is clear, the next step is the identification of one or more possible source substance(s). In many cases, the first approach is based on expert judgment derived from observation of the chemical structure. In comparatively easy scenarios, the source compound is a stereoisomer of the target compound or a salt with a different counterion or a substance sharing the same scaffold with minor differences in molecular weight. In other cases, the selection of source compounds is not trivial and requires the support of reliable computational tools that may assist in the identification of the proper chemical or group of chemicals. For this, the structure of the target substance should be transferred into a notation for molecular descriptions, for instance Simplified Molecular-Input Line-Entry System (SMILES) strings or the IUPAC International Chemical Identifier (InChI) keys, which are typically used as input into software for the calculation of molecular descriptors and physicochemical properties as well as molecular modelling (Heller et al., 2015). Some *in silico* methodology, e.g., docking simulations or molecular dynamic simulations, may require three-dimensional structural information (Luechtefeld and Hartung, 2017). The computational tool most frequently applied in the area of RAx is the OECD QSAR Toolbox^16^, which represents a good starting point for the identification of possible source substances. The QSAR Toolbox^16^ profiles molecules according to their chemical structure and corresponding possible reactivity in order to define criteria for category formation and provides the basis to explain the presence/absence of hazards or other properties.

After collecting candidates for source substances, the next step is the collection of all available information to fill the data gap and demonstrate similarity. A possible source compound is useless if there are no data or the available data are not accessible for any reason, such as the study report is proprietary or simply not available. Furthermore, existing information needs to be categorized and, in particular, all data should be analyzed to understand the mechanistic knowledge that they may bring along in order to identify biological pathways responsible for the outcome of an adverse effect that can be combined to substantiate the mechanism of action of target and source substances. If the mechanism behind an endpoint is unknown, chemical similarity and computational tools may still assist the hypothesis, but in this case, the prediction will be associated with a higher degree of uncertainty.

After the identification of possible source substances, and collection and categorization of the existing information, the next step is the formulation of a possible RAx hypothesis. Its confirmation may need the identification of a subset of assays and experimental tests to confirm the overlap of biological behavior through the demonstration that relevant key events are fully shared or that a regular trend exists (Zhu et al., 2016). In fact, evaluation of chemical similarity with Tanimoto metrics or other systems is useful but often not sufficient to demonstrate that the target and the source share similar properties for the endpoint under consideration (Mellor et al., 2019). For both regulatory acceptance and scientific reasons, it is preferable to broaden the perspective to include biological similarities and ensure that all possible source substances are taken into proper consideration.

The starting point of a strong similarity justification is the definition and characterization of all relevant physicochemical properties. This goal is achieved with the aid of available experimental information or reliable *in silico* predictions for both the source and the target substance(s). The full set depends on the specific substance and the *in vivo* endpoint under investigation, but should include as a minimum:

- Water solubility;
- Melting and boiling point;
- Octanol/water partitioning (K_o/w_);
- Volatility, e.g., as vapor pressure or Henry’s law constant;
- Particle size for powders;
- Stability (e.g., in air, aqueous solution, pH, light).

Other properties should be considered based on the molecular structure and the endpoint in question. For endpoints such as (DNA-reactive) genotoxicity or skin sensitization parameters, estimating the electrophilicity of certain functional groups or, more generally, the chemical reactivity might be helpful. Other useful further parameters could be, for example, chelating power, surface tension, oxidizing properties, and so on. The scope may include data from related areas, such as environmental fate data, in particular ready biodegradability, because they are indicative of a possible biological similarity, even though not directly relevant to the endpoint of interest. If not possible or too costly to obtain, there are programs that can provide relatively accurate estimations based on quantitative structure-property relationship (QSPR) models. Most of them are collected in the ECHA guidance for the preparation of REACH registration dossiers in the sections on adaptations of the standard testing regime (ECHA, 2017g). Others have been recently published, e.g., the OPEn structure-activity/property relationship app (OPERA; Mansouri et al., 2018) developed jointly by EPA and NICEATM and applied to more than 800,000 chemicals to produce freely available predicted properties on the EPA CompTox Chemicals Dashboard^18^. However, as these parameters are fundamental for predicting the biological activity of any substance, it is recommended, in general, to measure physicochemical properties with standard experimental protocols rather than to use prediction models, if possible.

When all available data are collected, they must be evaluated to decide whether they provide a clear picture and enough information to conclude whether the comparison between the source substances and the target substance is accurate enough to permit a conclusion and fill the gaps as required. If the approach seems reasonable but not sufficient to demonstrate the similarity, further tests on the category are necessary. The necessity to perform new *in vivo* tests should be considered at the very end of the process and only when all other opportunities have been considered. Gaps or uncertainties should be filled with existing studies or new *in vitro* tests.

Computational tools assist the identification of source materials and the demonstration of similarity, which would ideally include both toxicokinetic and toxicodynamic (TK/TD) considerations. Computational systems can be applied to analyze the issue globally, for example by defining the bioactivity profile through fingerprint description to feed machine learning models (Sturm et al., 2018). A useful database that can facilitate read-across groupings based on chemical similarity is the Danish QSAR database. It contains information on more than 600,000 chemicals from over 200 QSAR models^19^. Where models are used, they should be run also for category members for which experimental data exist. In this way, the applicability of the QSAR/QSPR model(s) to the specific set of chemicals under examination can be demonstrated. Where available, study results should be compared with the output of the computational approach as a reference to assess the accuracy of the prediction and claim better reliability for the output when applied to fill a data gap. Another computational tool developed specifically for RAx purposes is EPA’s Generalized Read Across (GenRA) add-in within the CompTox Chemicals Dashboard^18^, which uses a similarity-weighted activity algorithm to compute a RAx prediction using both chemical (structural fingerprint) and biological (*in vitro* HTS data from the Tox21 program) descriptors.

Given that potential selection bias is one of the major issues with respect to the reliability of the RAx approach, it is essential to document selection and deselection as well as chemical and biological characterization of the source chemicals that have been performed according to a clear rationale and in an unbiased way. Whatever the amount and quality of existing information, there will always be some uncertainties that need to be characterized, quantified and accepted. Computational methods may be very useful in providing variance and correlation among features. Uncertainty should be defined for each piece of information and in case of discordant results for the same substance, a conclusion should be drawn carefully by weighing all available information and relevant differences in the experimental protocol of the studies. Missing data and the lack of mechanistic coverage should be overcome with new *ad hoc* experimental studies.

### 2.2 New approach methodologies (NAMs) and adverse outcome pathways (AOPs)

**Fig. 4:**
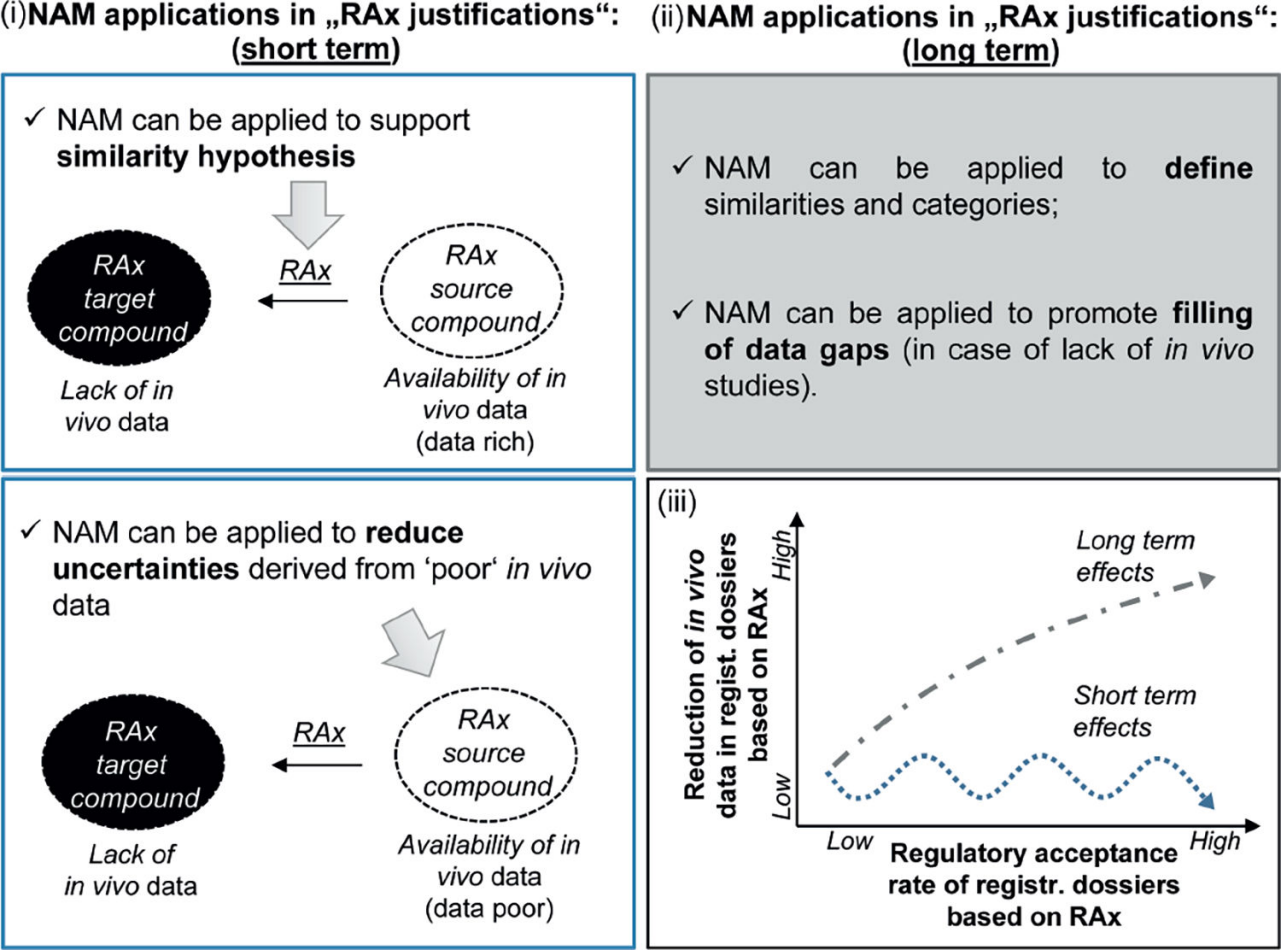
Inclusion of NAM-derived data in RAx justification dossiers to build confidence in its regulatory use (i) A registration dossier based on RAx always contains a justification including data that prove the validity of the similarity hypothesis and/or the reliability of the *in vivo* studies performed on the source compound. Recently, NAM-derived data have started to be used as complimentary information to support the RAx hypothesis. (ii) An increasing confidence in the use of NAM-data may lead to their exclusive use for similarity definition, category formation, and to fill data gaps (in case of lack of *in vivo* data). (iii) As short-term effect, the use of NAM-derived data in RAx dossiers can increase the regulatory acceptance rate of RAx justifications in risk assessment. As long-term effect, the increasing confidence in such data could lead to a RAx application based only on *in vitro/in silico* data.

As already mentioned, NAMs represent a good opportunity to support the RAx hypothesis by providing data to confirm whether a group of substances shares the same biological mechanism or if they show a specific trend within the category (Fig. 4). In the latter case, NAMs can help in the identification of the most representative compounds for further testing and may also contribute to the definition of the boundaries for the group. In this sense, NAMs should constitute new experimental tests/predictions that are performed with the specific goal of demonstrating the RAx hypothesis. The strength of NAMs in RAx is that all members of the category are tested simultaneously with the same test method, and the results are assessed as a category, demonstrating similarities and dissimilarities or providing clues to link the chemical structure to the biological activity.

A plethora of *in vitro* and *in silico* techniques are now available and can assist in the purpose of demonstrating similarities within a category. Listing all the opportunities is out of the scope of this paper, and more details are available elsewhere (e.g., Berggren et al., 2015). NAMs are often organized to model a key event (KE) of an AOP, the principle of which is to describe a sequential chain of causally linked events at different levels of biological organization that lead to an adverse health or ecotoxicological effect (Ankley et al., 2010; Stiegler et al., 2011; Becker et al., 2015; Dreser et al., 2015). The AOP concept is very similar to the pathway of toxicity (PoT) concept (Kleensang et al., 2014). AOPs provide a useful framework for comparing two or more chemicals and also a starting point for building the experiments for the biological demonstration of similarity (Nelms et al., 2018). In the definition of an endpoint, NAMs can be organized in an IATA that represents how the different approaches are combined within a regulatory context to reach a final conclusion on the hazard characterization of a substance^20^ (Leist et al., 2014).

The idea to apply the AOP principle to organize information was first proposed during a workshop at the OECD organized in 2010 (OECD, 2011). Members of a category should share the same molecular initiating event (MIE) and the same metabolic pathways (Cronin and Richarz, 2017). According to these authors, the role of AOPs to support RAx can be summarized as:

1. A plausible and transparent means of linking MIEs to the *in vivo* outcomes of regulatory interest and making uncertainties explicit.
2. A qualitative means of establishing causal linkages.
3. A conceptual framework for organizing information at different levels of biological organization, characterizing the WoE.
4. Evidence supporting the robustness of chemical categories.
5. A means of forming categories based on both intrinsic chemical and biological activity.
6. A basis for testable hypotheses, which in turn leads to the development and use of *in vitro* databases for developing new profilers and to establish response-to-response relationships.
7. A means of developing and justifying targeted and efficient testing and assessment scenarios that save time and resources, e.g., by identifying data gaps.
8. A means of supporting assessments of combined exposure to multiple chemicals within and across AOPs.
9. Greater biological context to what is currently a statistically based approach.

Even though AOPs, as presented in the AOPwiki^21^, incorporate a useful framework for comparing two or more chemicals and also a starting point for building the experiments for the biological demonstration of similarity (Nelms et al., 2018), sharing the same AOP may only demonstrate the possible biological activity but says nothing about whether this effect really occurs and does not provide an indication of the doses that may activate the effect, which is fundamental in chemical risk assessment.

**Fig. 5:**
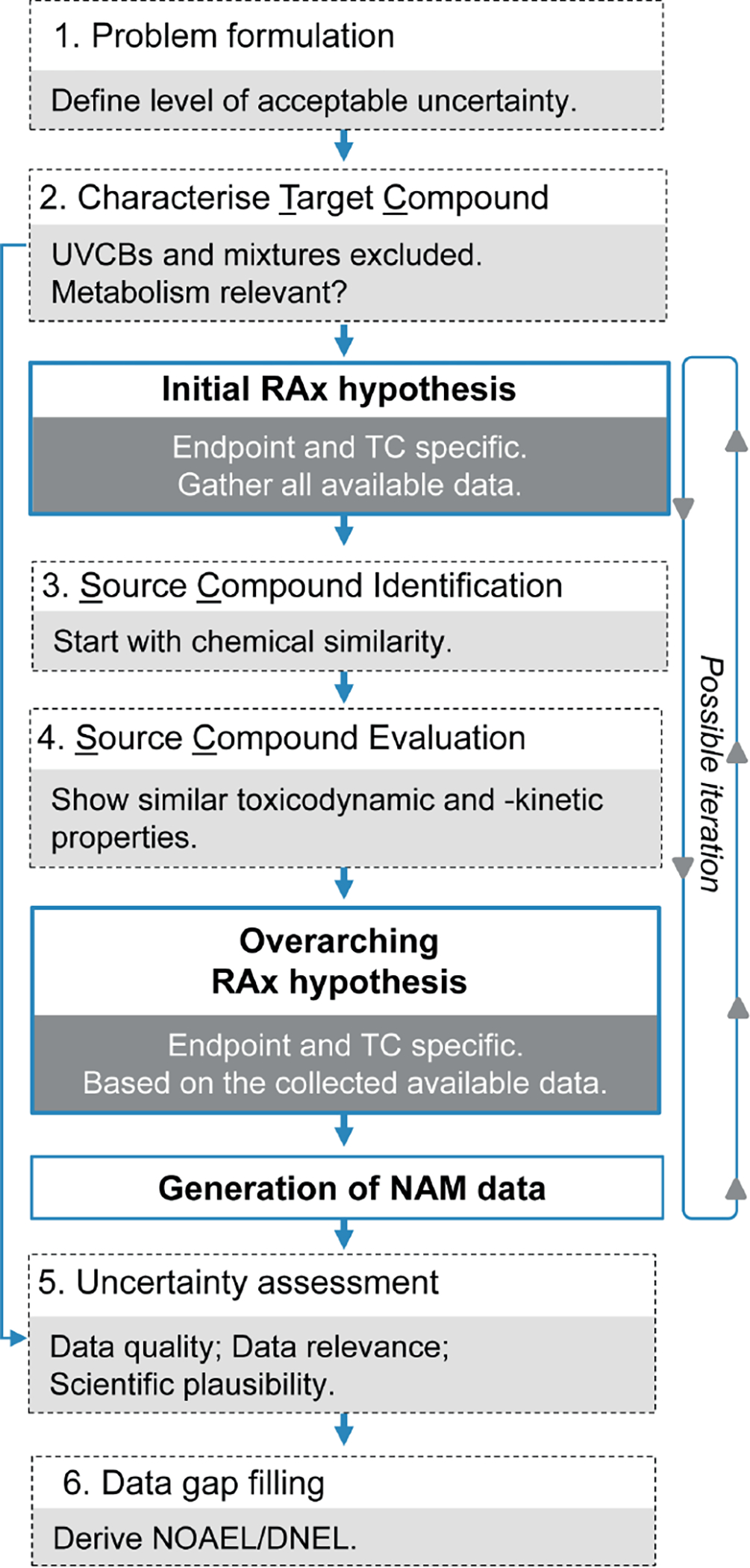
Workflow describing the RAx framework developed and applied within the EU-ToxRisk project

The EU-ToxRisk Project is developing conceptual frameworks to integrate NAMs for RAx assessment (Escher et al., 2019) and in particular on how NAM testing can prove the RAx hypothesis (Fig. 5). This concept has been developed through learnings from several case studies in which *in vitro* and *in silico* models are used to support the analysis of TD/TK properties, with a strong link between grouping and AOPs.

In the future, NAMs in combination with RAx represent a strong possibility to dramatically reduce or even eliminate the need for new *in vivo* testing. This will happen when the development of *in vitro* testing is mature enough to fill the data gaps in the categories (Fig. 4).

### 2.3 Absorption, distribution, metabolism and excretion (ADME)

ADME profiling represents an important step of RAx justification and, in particular, for moving from hazard to risk assessment. ADME also can be useful to select source substances.

To complement this information, both the source and target substance need a proper characterization of ADME data (Tsaioun et al., 2016). Unfortunately, outside of the pharmaceutical and agrochemical sector, this information is at best partially available from dedicated experimental studies.

Differences in absorption rate and distribution can be considered in RAx for risk assessment purposes, and big differences may be considered a pointer towards more important dissimilarities between chemicals, while the demonstration of similar absorption and distribution may support the RAx justification. Information on the distribution of a substance in the organism can also indicate a possible target organ. Here NAMs can be supported by quantitative *in vitro* to *in vivo* extrapolation (QIVIVE), even though there are still no valid and universally applicable systems (Hartung, 2018; Kisitu et al., 2019). Excretion mode and kinetics represent other parameters affecting systemic uptake and exposure, and they should always be considered to conclude on the similarity among the components of the category.

Metabolism is one of the most important steps of the ADME process. RAx studies where the source and target compounds share the same active metabolites are an infrequent but fortunate situation, allowing an easy justification of the RAx approach (van Ravenzwaay et al., 2016). In general, dissimilarities in metabolism will considerably complicate the RAx, since this would trigger the need to demonstrate the absence of (relevant) toxicity for all metabolites not common to both source and target chemicals. Metabolism assessment can be performed through a new experimental *in vitro* study, for example with primary hepatocytes, which can provide an idea of clearance and transformation. Unfortunately, an exhaustive analysis of metabolism requires long and expensive studies, and it is hardly ever performed outside of pharmaceutical and agrochemical applications. To cope with the lack of experimental data, chemoinformatics may help to provide a prediction of metabolism products (Madden and Cronin, 2006; Kirchmair et al., 2015; Djoumbou-Feunang et al., 2019; Miller et al., 2019). Computational metabolite predictions represent a valuable tool that may identify outliers in a group of similar substances or explain differences when *in vivo* animal studies are applied to the situation in humans.

The EU-ToxRisk project is taking up the challenge of ADME, being aware that, in many cases, lack of demonstration that target and source substances are similar was the cause of rejection of the RAx justification by regulators (Ball et al., 2016). The RAx is acceptable only if sufficient similarity in ADME properties between source and target chemicals is demonstrated and the possible impact of any dissimilarities is sufficiently explained. The absence of reliable ADME information often represents one of the weakest points in RAx justifications, and in this regard it seems fair to note that by not making ADME data a mandatory standard requirement, the REACH Regulation has put a major hurdle in the way of achieving its own goals in terms of facilitating the move away from *in vivo* animal testing.

### 2.4 Applicability domain of RAx

The ECHA RAAF document is very explicit in limiting RAx to a single endpoint (ECHA, 2017b,c). On the other hand, the success of RAx depends on a more holistic approach facilitated by the analysis of large quantities of data. Even though the final goal is the prediction of the specific endpoint, this always should be connected to a more general analysis of all possible analogues and groups of chemicals.

Enoch et al. (2008) demonstrated that reliability of QSARs is improved when modelling the individual mechanisms of toxic action present in the data set. The link of the prediction to the full elucidation of the mechanism provides specific limits to the prediction, with a precise definition of the boundaries for the group of chemicals in RAx, including the applicability domain. The one-to-one comparison, i.e., one target and one source chemical, should be applied only in case of very strong evidence of similarity and identical biological activity. Rather, any demonstration of similarity should benefit from a global analysis that includes not only a larger set of chemicals but also the assessment of general toxicity of the substances, with a possible explanation of the differences that may derive also from physicochemical properties.

The concept of local validity (Patlewicz et al., 2014) can be applied in reverse mode. The explanation of a possible mechanism can set the boundaries for the applicability domain of RAx by imposing the use of a specific method to substances that show a specific mechanism or that are metabolized to a specific chemical.

The mechanistic applicability domain is one important aspect, but the structural and physicochemical descriptors, as well as property and effect spaces also need proper attention when building a category. Overall, the applicability domain of a RAx category can be considered a multidimensional space composed of all these elements, in which members of a valid category are located relatively “closely” to each other. In case of even distribution of the category members, trust in RAx categories is increased if the category encompasses a densely populated multidimensional space and the target compound is located in such an area.

### 2.5 RAx for non-classified substances

Declaring the existence of a certain risk of toxicity for a substance is comparatively easy, even though there is always the possibility of a hidden risk of higher concern. The situation is different when the RAx exercise aims to demonstrate the absence of concern, because this conclusion should really include the demonstration that any possibility of hazard has been taken into account. For this reason, RAx for substances with no or low activity requires more comprehensive justification, supported by a high level of confidence and strong evidence. At the same time, most chemicals do not seem to require hazard classification (Hoffmann and Hartung, 2005; Luechtefeld et al., 2016a), and therefore it may be expected that the number of RAx resulting in a “negative”, i.e., absence of relevant effects, outcome will be clearly higher than that of RAx predictions with a “positive” effect.

The big question is how to achieve sufficient confidence in negative conclusions on toxicity. It is a truism that scientific knowledge develops over time, and the regulatory landscape will continue to change based on the discovery of new or a re-evaluation of known mechanisms and effects of chemicals in biological systems. Therefore, the natural answer to the above dilemma could be that predictions of a general absence of toxicity should cover all areas of potentially adverse effects known and considered relevant in the respective legislative framework at the time the prediction is performed. Under REACH, for example, the range of endpoints to consider in general is characterized by the output of the methodology accepted for the generation of the standard information specified in Annexes VII-X, even though it should be enlarged in situations where there is a reason to assume that the standard information is not sufficient. As a consequence, confidence, whether in traditional assessment or RAx, is best increased by considering a wide range of endpoints with in-depth TK/TD analysis. In the scope of RAx, the only way to demonstrate that a specific substance activates no toxicological alert should be supported by either a higher number of source substances, all demonstrating biological inactivity, or a strong demonstration of similarity if there is only one source substance. As far as possible, the latter case should always be supported by a further demonstration that “biological surprises” appear unlikely, for example by providing evidence for similar substances. A way to tackle uncertainty is by using a set of independent information that leads to the same conclusion in a WoE approach (Linkov et al., 2015). Schultz et al. (2017a) have provided a good example with the analysis of a set of n-alkanols, which were analyzed by grouping substances sharing common absorption profiles and metabolic pathways, confirmed by the existence of good 90-day oral repeated dose toxicity studies in rats.

In conclusion, general rules for RAx validity are applied also for demonstration of the absence of biological concern, with a further detailed description of ADME and all possible MoAs/AOPs that may occur. In the future, omics profiling or high-throughput testing schemes such as the Tox21/ToxCast program might aid in demonstrating a lack of biological activity over a broad range of effect markers.

As reported in other sections, the amount of information considered sufficient depends on the risk that is related to the assessment together with the regulatory application scope and should be considered on a case-by-case basis. The principles described to reduce the uncertainty are generally valid, but they should be applied even more rigorously for the definition of the absence of concern. More specific guidance would be helpful here.

### 2.6 Hazard characterization and potency

A comparatively easy application of RAx is hazard identification by means of a demonstration that two or more chemicals may qualitatively exhibit the same toxicological effect. In contrast, the prediction of potency, e.g., in the form of a classification sub-category, places considerably higher demands. Hazard characterization is communicated to all users with specific pictograms and hazard phrases, as internationally agreed by the United Nations in the Globally Harmonized System of Classification and Labelling of Chemicals (GHS), which was implemented in the EU with the Classification Labelling and Packaging (CLP) Regulation (EC) 1272/2008, which explicitly accepts RAx when describing the scope of WoE determination in its Annex I.

The difficulty in applying RAx for classification purposes lies in the analysis of potency for the determination of hazard category. Categories of local effects are generally well characterized with defined thresholds, while more concerning endpoints, such as CMRs (carcinogenic, mutagenic and reprotoxic substances), have no defined threshold in CLP, and the category is demarcated only by the qualitative probability that a substance may exert that effect. In fact, Category 2 is based on the presumption that the toxicity outcome may result, Category 1B is the proven toxicity in animals, and Category 1A the proven toxicity in humans. In all cases, there is a need to assign a dose to the hazard, which is a defined threshold for local and acute toxicity endpoints and the no-observed-adverse-effect-level (NOAEL) for the systemic endpoints, for risk assessment.

The OECD guidance on grouping of chemicals provides some consideration on how a quantitative prediction for target substances can be made by extra- and interpolation within a category for which trend(s) in toxicity or factors influencing toxicity have been identified. When the AOP and quantitative information on the relationship between KE and AO are known, NAMs can be used for the target and source substances to further strengthen the (quantitative) prediction, with the application of a suitable QIVIVE correction. While larger categories might provide the basis for trend analysis and thus a relative potency assessment of their members regarding a specific effect, the situation is much more difficult in one-to-one assessment, and additional reasoning will generally be required to assign the same, or a higher or lower, potency to the target. Depending on the legislative framework, the assignment of additional assessment factors (AF) might be required in such circumstances. A more scientific approach could consider a detailed toxicokinetic assessment with correlation of the exposure and the concentration at the target site.

This particular topic clearly needs more discussion and agreement between regulators.

### 2.7 Practical case studies

The published literature gives an indication of the considerable breadth of RAx studies that have been undertaken and reported. Whilst this is not necessarily representative of all REACH submissions, it does indicate that RAx has been applied in a number of scenarios and with a variety of techniques. Turning some of these into case studies is useful for a number of reasons that go beyond simply providing an illustration for further use (Tab. 1).

For the illustration of RAx in various forms, the OECD has published case studies on the applicability of IATA including RAx (OECD 2016b, 2017b, 2018). These examples perfectly demonstrate the new idea of risk assessment as a combination of QSAR, RAx, NAMs and WoE. The way they are presented is not intended to provide exhaustive documentation to justify the RAx approach, but rather to analyze the state-of-the-art of this methodology, with particular attention to strengths and pitfalls. The focus is on one or more specific endpoints such as mutagenicity, repeated dose toxicity, etc. and not so much on the categories themselves.

As part of the SEURAT-1 initiative, RAx case studies were instigated (Berggren et al., 2015). This resulted in the development of a template for reporting RAx (Schultz et al., 2015) as well as the publication of some of the findings in the peer-reviewed literature (Schultz et al., 2017a,b; Mellor et al., 2017). Analysis of the case studies overall allowed Schultz and Cronin (2017) to provide practical examples on how to reduce the uncertainty related to the RAx assessment, concluding on the importance of data quality, similarity argumentation and justification, and the overwhelming need for TK/TD data (Pamies et al., 2018). These analyses later influenced the description of the overarching uncertainties associated with RAx, allowing for a series of questions to probe the quality, or otherwise, of the underpinning science and data (Schultz et al., 2019).

The EU-ToxRisk Project has started an initiative in collaboration with regulators from different scientific areas, including experts in the areas of pesticides, food safety, industrial chemicals, etc. Partners have prepared mock submissions to regulators from agencies associated with European regulations in order to learn whether their approaches would be acceptable in a legal context or not. In this way, EU-ToxRisk case studies are treated as if they were real cases with the purpose of challenging the traditional regulatory assessment approach. A dedicated workshop with the active collaboration of ECHA and EFSA has already taken place^22^, and the final report should be published in 2020.

In recent years, also ECHA and the EU Member States have increasingly included grouping approaches into their screening and assessment schemes. In particular, ECHA launched the collaborative approach (COLLA) pilot projects focused on five groups of substances (ECHA, 2018). The aim was to improve the information used to decide on the needs for further regulatory risk management with the involvement of Member State competent authorities and concerned registrants. The projects explored how the overall grouping approach can be used to clarify and address the identified concerns and what type of supporting information is required. The projects tested two different elements: addressing substances by groups and early interaction with registrants. It was reported that the resources spent by ECHA and Member State authorities were significant, and almost equally divided between the screening and the interaction phases. The COLLA could demonstrate an increase in effectiveness, providing a better picture of the gaps and the points for concern, while it was difficult to conclude on benefits in efficiency due to the amount of resources required in the management.

In the scope of REACH, other simple approaches have been used in the area of metal salts, e.g., for nickel, cobalt, iron chlorides, sulfates, nitrates, etc. More complex RAx categories have been built for petroleum/coal products, fatty acid derivatives, etc. With the exception of a few publications (Clark et al., 2013) or indications on the consortia website such as CONCAWE^23^, these data mainly derive from the personal experience of some co-authors and are generally not public. Further analysis is not possible and, moreover, the general feedback from the authorities is not known, unless looking for data relative to the submission of the individual substances. Even though it would be interesting, this search is out of the scope of this publication.

### 2.8 Integrated use of RAx

The main potential of RAx lies in its application in an integrated way on several source substances, so it is more than a simple tool to waive a single *in vivo* test. Rather, the assessment relies on a more global approach that considers a variety of data. Even if the comparison can be between one source and one target, the use in support of many other chemicals in a group or the comparison between other similar substances can be of benefit. Let us consider a very easy example of the RAx of a sodium salt (source) with its acid. If the dissociation constant confirms that the acid is fully dissociated at the physiological pH, such information already might seem enough to justify the read-across. However, the hypothesis would be strengthened further if there is the possibility to compare other experimental endpoints for both substances, highlighting differences that may derive only from the capacity of the acid to alter the pH. It would not be surprising if the acid was classified as an irritant and the salt was not, but they may share identical biological behavior with regard to mutagenicity, developmental toxicity, etc. RAx hypothesis supported by other similar substances, with data on both the acid and the sodium salt, and even other salts, would then appear much stronger.

**Fig. 6:**
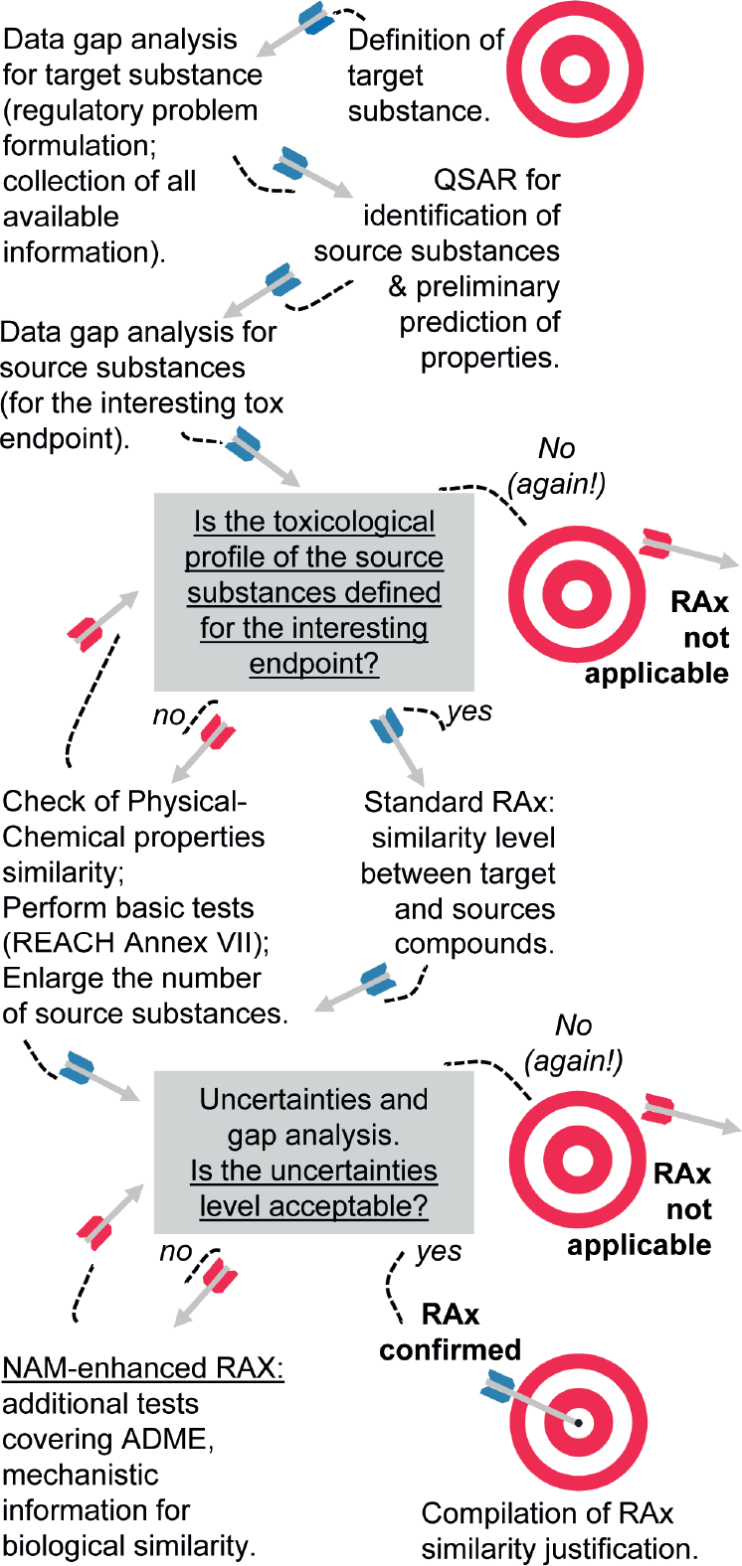
Decision strategy for the application of a RAx framework for risk assessment After the definition of the target substance and the identification of data gaps, the first step is the identification of all possible source substances with the support of QSAR tools. The process proceeds only if it is demonstrated that target and source substances share a similar toxicological profile. A full characterization of the physicochemical properties and the experimental determination of basic biological endpoints, such as those required in Annex VII of REACH, can support this step. The NAM-enhanced RAx benefits from the performance of suitable *in vitro* tests that can provide mechanistic information for biological similarity or elucidation of ADME. The whole procedure moves forward in an iterative way. On the other hand, if RAx is confirmed, the last step is the compilation of the RAx report that should contain all details of the steps and the decisions taken along the process.

**Fig. 7:**
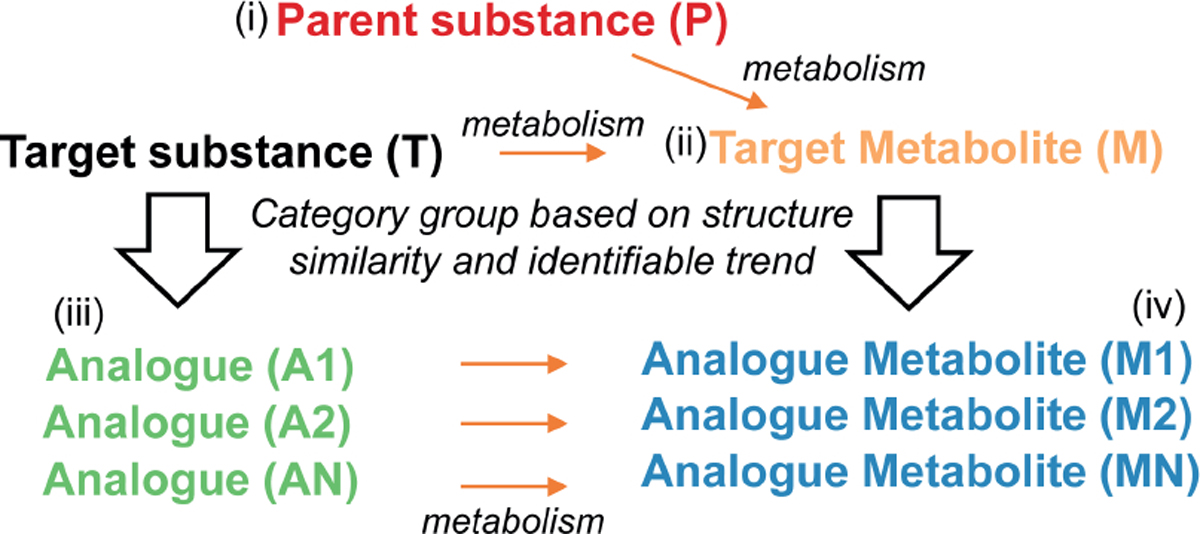
Scheme for the identification of possible source substances in a RAx approach Given the target substance T, possible source substances are (i) substances producing the same metabolite M (parent substance P) but also (ii) the metabolite itself (target metabolite M). This pattern can be confirmed if other substances present the same trend in a category, where substances A1, A2, etc. (iii) metabolize to give metabolite M1, M2, etc., which can also be grouped together (iv). Availability of existing *in vivo* studies may be random in such a scheme. Nevertheless, they may predict the possible effect of target substance T with a high level of confidence.

In a more complex situation, the application of RAx requires a holistic approach that considers a multitude of data gained from both the target and the source substances. In Figure 6, the process for the demonstration of similarity is described. Such process is based on:

1. Similar chemical structure and behavior;
2. QSAR prediction;
3. Collection and evaluation of existing *in vivo* studies;
4. Assessment of new *in vitro* tests to confirm the mechanism. If the RAx hypothesis is based on the hypothesis of the same TK/TD profile, strong RAx justification should include, if possible (Fig. 7):
  - Comparison between two substances sharing the same metabolic products;
  - Comparison with the substance representing the metabolic product itself;
  - Comparison with a group of similar substances showing a comparable metabolic trend.

From this perspective, RAx is much more than the comparison of two chemicals to waive an *in vivo* test, rather it is a complex system that studies toxicity in a highly integrated manner (Fig. 6). The final goal is an increase of knowledge on the toxicological profile of substances. This principle is well described in the EFSA document on WoE (EFSA, 2017). The EFSA guidance defines three steps for the WoE approach, i.e., assembling the evidence into lines of evidence of similar type, weighing the evidence, and integrating the evidence. The three key parameters are i) reliability, as the extent to which the information comprising a piece or line of evidence is correct, ii) relevance, as the contribution answering a specified question, and iii) consistency, as the compatibility of the different information towards the final toxicity prediction.

Regarding new *in vitro* tests, those should be tailored to the specific need, whether it is the demonstration of a shared KE within an AOP or a TK/TD route. Other tests should be considered, such as the fish embryo toxicity (FET) test, if there is an issue related to developmental toxicity (Kroese et al., 2015). The selection of the right method should consider the applicability domain, the regulatory scope, and also other practical constraints, such as experimental availability and costs, with the latter probably being the main obstacle to wider use of these new tools.

At the moment, this procedure is based mainly on expert judgment, but the inclusion of defined approaches, i.e., testing strategies with clearly specified information sources and objective data interpretation procedures, is highly recommended for the future in order to increase the objectivity of the final outcome and to guarantee that all available data and information is taken into consideration. The analysis of dossiers containing RAx justifications that have been rejected by the authorities can be very useful to understand the regulatory requirements of an application (Ball et al., 2016).

## 3 Regulatory use of RAx

### 3.1 Validation

Validation of the RAx approach represents the best opportunity for regulatory acceptance and the necessary step for reducing uncertainties and building scientific confidence. More specifically, method validation is a process designed to show that a given method is adequate and reliable for the purpose it was designed for. The requirement of validation is a direct consequence of the scientific method and addresses many of its fundamental aspects, e.g., hypothesis formulation, hypothesis testing, predictions based on hypothesis, replicability, bias, uncertainty, etc. If a method has not been validated, this means, in effect, that it is unknown whether it successfully serves the purpose it was designed for. The usefulness of such a method is then subordinate to subjective beliefs. This is a criticism brought forward also against the currently applied *in vivo* methods, most of which were never formally validated but which are still used in risk assessment following conventional scientific acceptance.

Regarding RAx, its validation should demonstrate that the approach leads to the expected result, e.g., that toxicological effects (or their absence) as observed in traditional *in vivo* or *in vitro* tests are correctly predicted within reasonable limits. RAx is not *per se* a defined method, and therefore it cannot be validated following the procedure that is traditionally applied for *in vitro* tests^24^. Also, each RAx is a case-specific assessment combining purely computational tools with a mix of chemistry, mechanistic knowledge, QSAR, *in vivo* and *in vitro* techniques, brought together under the auspices of expert judgement. Source and target substances can be unique or multiple, and the endpoint comparison can be based on the same value or following a trend. The quality and amount of source data are also variable. Nevertheless, the adequacy, reliability and relevance of individual test or prediction methods combined in a RAx argument can be assessed, as can the overall plausibility of the RAx justification along with the extent to which uncertainties remain.

In many cases, the RAx approach is performed manually by an individual, or group of, toxicologist(s) (Benfenati et al., 2016). Although standardized RAx workflows (similar to a “defined approach” (DA) as used by the OECD, i.e., a defined set of information sources assessed with a defined data interpretation procedure) or even automated RAx would appear a good option for providing an impartial output that is always repeatable and independent of the operator, fully automated assessment should not be run in an unsupervised mode. The best option should be somewhere in the middle, i.e., a standardized/automatic evaluation of particular criteria supporting expert judgement and evaluation providing performance indicators for expert review but not necessarily an overall opinion. Even then, the best diagnostic tools will have little value unless they are used by operators who are familiar with the RAx technique as well as the underlying toxicology and chemistry of the specific case and the context-specific criteria for scientific validity. For example, the most recent version of the OECD QSAR Toolbox^16^ supports the user by providing information on alert performance based on the category candidates that are selected (Yordanova et al., 2019). Recently, Luechtefeld and co-workers (2018a) attempted this task using an automated RAx-based QSAR approach on a huge set of acute and topical toxicity as well as ecotoxicological endpoints. They showed that on average a given self-classification in REACH registration dossiers could be reproduced with a RAx-based QSAR model based on the nearest neighbors of that chemical in descriptor space with higher reliability than was found for traditional test results in the REACH database. Notably, this work focused on a simple GHS/CLP classification endpoint, whereas such work currently cannot be realized for complex, multiple-endpoint systemic toxicities, even though some authors see such methodology as promising in the future, and this possibility should be kept open (Berggren et al., 2015).

Many aspects of RAx are subjective and difficult to quantify. For instance, the evaluation of similarity is very challenging, and minor differences that seem negligible may have a strong impact on the biochemical activity, as illustrated by the problem of activity cliffs. If there was agreement on suitable metrics and on minimum thresholds, this could be used as one of the parameters defining the applicability of a given RAx.

Moving to a possible practical approach, aspects of RAx adequacy, reliability and relevance can be analyzed, as already proposed for the validation of testing strategies (Rovida et al., 2014), by using the five OECD Principles established for the validation of QSARs (OECD, 2007). For the specific case of RAx, these can be interpreted as follows:

- *A defined endpoint*. RAx assessment should be performed for each endpoint for which sufficient data are lacking. Often there is the danger of considering the RAx globally, without a clear definition of the specific activity that it is required.
- *An unambiguous algorithm*. This has to be applied to all algorithms that are part of a RAx, e.g., those used for similarity assessment or for weighing information in the context of a WoE decision. The procedure for RAx should be clear, transparent and reproducible, i.e., different experts should arrive at comparable conclusions based on the same set of data.
- *A defined domain of applicability*. As noted above, in RAx the applicability domain is characterized by the structural, mechanistic and property space spanned by the category members, while the resulting prediction is subject to the boundaries defined by the regulatory scope of the requested assessment. However, this step should address also the adequacy of the available information. Proper characterization of the applicability domain will also help to identify potential bias in the selection or characterization of the tested substances.
- *Appropriate measures of goodness-of-fit, robustness and predictivity*. For the RAx, being an individual and case-specific assessment, performance statistics cannot be obtained, but this should be done for all non-standard methodology, e.g., *in vitro* and *in silico* NAMs, used to obtain the overall RAx conclusion (Bal-Price et al., 2018). Evaluation of robustness should include also the suitability and quality of the available information, e.g., if there is bias in the characterization of the tested substances.
- *A mechanistic interpretation, if possible*. Where available, mechanistic information is extremely helpful to strengthen the plausibility of a RAx, with NAMs possibly applied for that purpose.

These points should be addressed via a solid assessment of the available data, methods using pre-defined criteria, and rules that are standardized and harmonized on an international scale as far as possible. Subjective expert judgement should be avoided as much as possible; where it is needed, a set of transparent, pre-defined criteria and rules should be applied.

An opportunity to guide RAx validation starts from the definition of the different types of RAx that are applicable. The ECHA RAAF document (ECHA, 2017b,c) provides a good definition of the six possible scenarios: scenarios 1 and 2 refer to one-to-one read-across (analogue approaches), scenarios 3 and 4 to category approaches with variation in the properties observed among the source substances, and scenarios 5 and 6 to categories without such variation. The ECHA document explains in detail the basis for the RAx hypothesis that backs each scenario, with a list of pre-requisites that the submitter needs to fulfil for the acceptance of the correspondent RAx strategy. Based on these scenarios, assessment elements can be defined by constructing a set of questions that should be addressed to challenge adequacy, relevance and reliability of the RAx performed, in other words, its scientific validity. The answers given to those questions should arrive from a mix of computational systems and expert judgement, with the dilemma in setting the border between the two.

The definition of a case study that is assessed by different teams to refine the approach until the same conclusion is shared may represent a good exercise for validation. The main difficulty of this approach is that it is well known that the conclusion on risk assessment is already different when performed by different experts even if the evaluation is based on the same set of *in vivo* studies (Ruden et al., 2001). Quality of source data is also a fundamental parameter to be considered in a validation process. A number of quality scoring tools are available for this goal (Samuel et al., 2016).

Validation is useful only if universally recognized, in different countries and by different agencies, considering that the regulatory endorsement is necessary for final acceptability. The challenge in this sense is not restricted to the validation of RAx. In many cases, there is a lack of consistency between different assessors. Bearing in mind that at this stage a global validation of RAx is practically impossible, efforts should still be made to harmonize the approach and improve regulatory acceptability.

As part of the GRAP, good reporting is an essential pre-requisite for a suitable assessment of adequacy, relevance and reliability (Hartung et al., 2019). The final report should include all essential information on how the assessment was performed, with a clear indication of the parameters that were applied. The assessor should be capable of repeating all the steps that were performed and control possible sources of bias and errors. Schultz et al. (2015) have prepared a comprehensive analysis of the main elements that should be included in a RAx report, with particular emphasis on the importance of a good organization of the data to increase confidence and acceptability (Leist and Hengstler, 2018). Automated systems with their reporting templates may offer advantages here.

### 3.2 Uncertainties

Even though part of the validation process, evaluation of uncertainty deserves a dedicated section. In fact, it represents a key step to increase confidence and improve regulatory acceptability, even though often disregarded in the validation processes. Assessment of uncertainty and confidence in RAx prediction should be a specific element of the RAx workflow. A defined and unique method to assess uncertainty does not exist. However, several approaches and frameworks have been proposed, with subjective and “expert-driven” methods, i.e., relying on expert judgment to evaluate the relevance of the analogues, their underlying data and the whole RAx argument (Blackburn and Stuard, 2014; Schultz et al., 2015), or by proposing more objective quantitative approaches for assessing uncertainty (e.g., Shah et al., 2016). There is no “best” approach, especially considering that each RAx study should be assessed on a case-by-case basis, depending on the different scenarios, the hypothesis, and the rationale. However, it is important to follow a structured framework and to analyze and combine all the uncertainties for each step/element of the RAx workflow to finally derive overall confidence in the RAx prediction. Qualitative ranking (e.g., low/medium/high) may be helpful, but the final impact should be carefully weighed. Automated RAx approaches have the advantage that a certainty of prediction can be assigned depending on the available source data quantity and quality, heterogeneity, and degree of similarity to the target structure (Luechtefeld et al., 2018a).

Uncertainty is embedded in all scientific measures and decisions. For traditional *in vivo* toxicity studies, this has been addressed lately (WHO, 2018) but not yet solved, the report making the following observation “*the evaluation of uncertainties in risk assessment is a crucial issue, with direct consequences for risk management*”. EFSA (2018b) recently published a key document on uncertainty analysis in risk assessment defining the uncertainty in toxicological assessment as “*all types of limitations in available knowledge that affect the range and probability of possible answers to an assessment question*”. A first distinction is made between uncertainties associated with assessment inputs and those associated with assessment methodology, where both are applicable to the case of RAx. Regarding the acceptable level of uncertainty in light of the consequences, it is interesting that in addition to the possible impact of the final decision on, e.g., health and economic factors, the urgency of a decision regarding the final goal of the risk assessment might also need to be considered. Emergency assessments, e.g., after a spill of chemicals, sometimes have to favor fast methods over more reliable and sustainable ones. The EFSA Guidance comprehensively describes a methodology for identifying, assessing, describing and, in some cases, quantifying uncertainty, providing some general principles applicable in the evaluation of RAx. Results must be expressed in an understandable way, but the consumers of these results need to be receptive.

Frameworks such as the Dempster-Shafer theory allow for the combination of uncertainty from diverse sources and lead to reasonable hazard estimates (Dutta, 2015; Rathman et al., 2018), even though other methods are also outlined in EFSA (2018c).

**Fig. 8:**
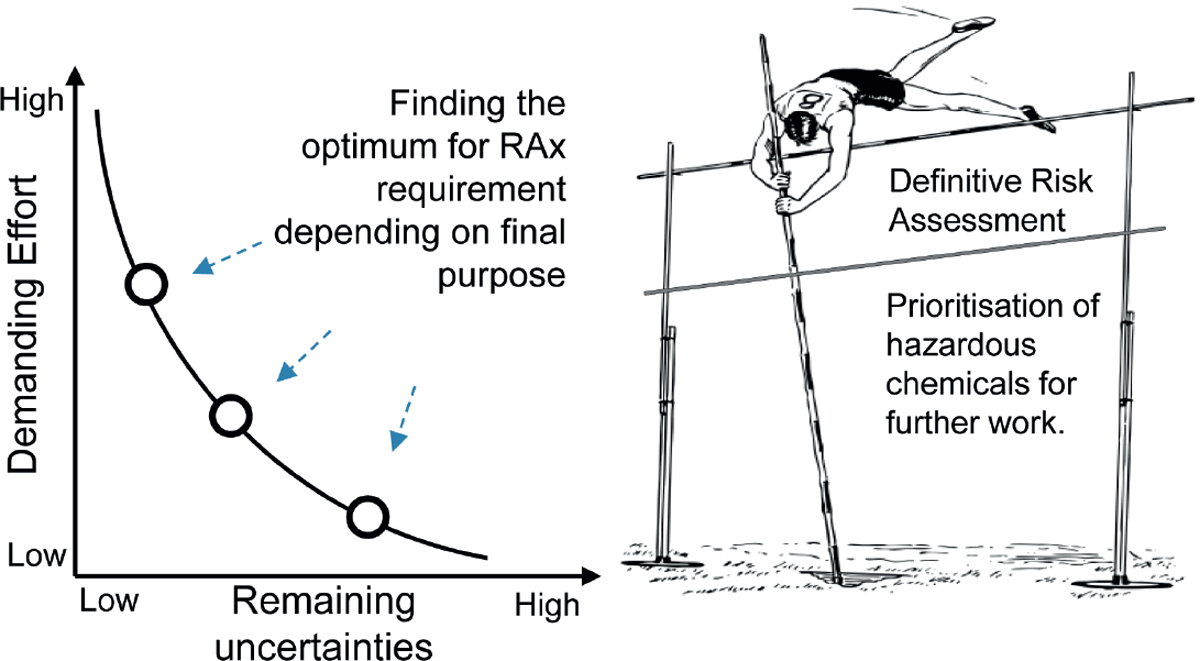
Tailoring the threshold of acceptable uncertainties based on RAx purpose Different thresholds of acceptable uncertainties even though there are exceptions. In some situations, we may accept a high level of uncertainty in risk assessment if the margin of exposure (MoE) is large. Regulatory use of the RAx approach strongly depends on the regulatory framework for which it is used. The purpose will also define the acceptable level of uncertainty. For hazard identification and for the classification and labelling of a systemic effect, the acceptance may depend on the accuracy with which an effect within a toxicity area can be determined. In risk assessment, the demand on the acceptance of the uncertainty is higher.

The regulatory use of the prediction and, in general, the final aim of the risk assessment should provide the level of uncertainty that is acceptable (Fig. 8). The process of substance prioritization, on the one hand, is probably the least demanding, as it is necessarily followed by further testing. On the other hand, the evaluation of a cosmetic or a food ingredient with widespread use among consumers requires much more care. If it is in the scope of a specific regulation, acceptability limits in the legislation text should be analyzed in detail.

Schultz et al. (2019) reviewed six previously reported case studies with the aim of identifying uncertainties that potentially impact the acceptance of a read-across prediction. They identified a total of twelve sources of uncertainty and formulated thirty questions to assist in assessing uncertainties, as reported in Table 2. The answer to each of those questions may guide the applicants in the analysis of the uncertainty of the approach. This process needs a very robust approach to demonstrate objectivity and transparency (Schultz et al., 2015).

In the context of RAx, a major issue in terms of uncertainty relates to the identification of source substances. This step should follow strict rules to guarantee that any available source is considered, with no exclusion based on the “cherry-picking” principle. Demonstration of a systematic and extensive search for analogues by means of a computer system may increase the reliability of the operation. Using a worst-case compound in a RAx scenario may help to reduce the uncertainty that the outcome could underestimate the risk. Another aspect of RAx uncertainty is linked to the endpoint gap that the RAx should fill. Some endpoints are characterized by a well-defined mechanism and, as a consequence, the possibility of finding suitable source materials is easier. In contrast, for endpoints such as repeated dose toxicity, carcinogenicity or reproductive toxicity, the sources of uncertainty are broader and less quantifiable. The quality of source data and the unknown impurity profile of the test item is another source of uncertainty. Uncertainty of RAx applied to UVCB substances is more challenging but still controlled if the evidence allows identification of the constituents representing the main drivers of toxicity (EFSA, 2019). In case of high batch-to-batch variability in the composition of the UVCB substance, NAMs can provide high-throughput test arrays and omics methods to characterize the differences and guarantee the absence of possible concern.

For consistency of data, the risk to underestimate contradictory data or even exclude undesirable results is very high, and this is why the application of automatic tools is necessary to increase the confidence in an unbiased final conclusion. Careful evaluation of the available data is fundamental but often difficult when the source study reports are not available.

**Fig. 9:**
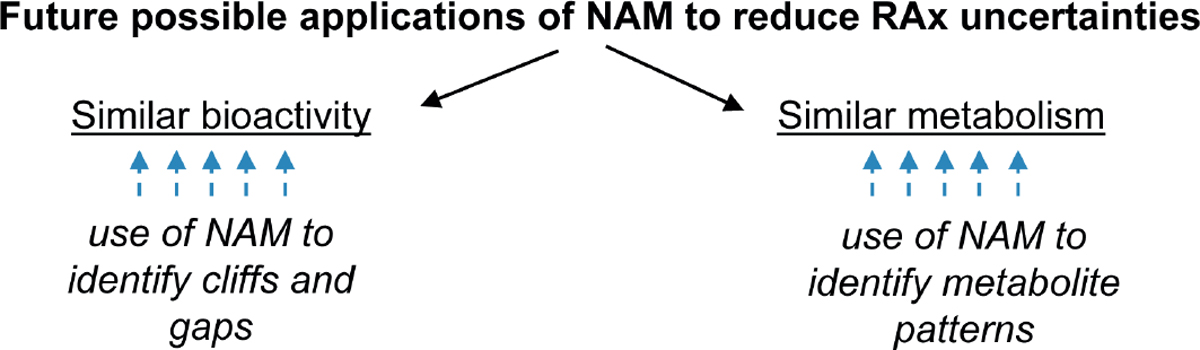
Applications of NAM to reduce uncertainties in RAX justifications The same *in vitro* test is performed on all substances belonging to a category to demonstrate the similarity and identify possible activity cliffs. New *in vitro* methods are also used to identify metabolite patterns and to assess whether they are shared among the components of a category.

Once finished with the analysis of available information, the following step is the justification of the similarity. While several tools are available to quantitatively compare two or more substances from a chemical point of view, this is not the case for the comparison of similar biological mechanisms, which nevertheless is highly desirable, even though it requires deep knowledge of the biological mechanism underneath the adverse effect. The level of uncertainty can be further decreased by performing new experimental studies. Sometimes additional experiments might need to be carried out to determine the difference between two substances and to measure the trend of a specific property in a category, identify an activity cliff or provide bridging information, e.g., for QIVIVE (Fig. 9). This can be useful also to define the boundary of the category more precisely. The addition of information on physicochemical data can support the hypothesis (Helman et al., 2018) and highlight the presence of activity cliffs that may represent a serious source of errors. Estimation of metabolism is particularly important and should be performed very carefully, considering also that for the comparison of source and target substances, metabolism represents a means of similarity justification. It is generally difficult to assess the quantitative relevance of predicted metabolites. Even though there is no way to exactly quantify uncertainty in manual RAx, extensive justification following the flowchart of Table 2 can help in the justification of similarity, even in case of absence of effects whose demonstration is always more difficult. In conclusion, uncertainties can be reduced by appropriate and directed testing with a focus on TK/TD and the use of NAMs to support similarity justification, in many cases proving the same hazard class.

### 3.3 Good Read-Across Practice (GRAP)

GRAP represents an important opportunity to increase the regulatory acceptance of RAx by improving scientific plausibility. Ball et al. (2016) summarized the main steps to establish GRAP, starting from the collection of REACH registration dossiers containing unsatisfactory RAx presentations. The most significant factor for rejection was the lack of sufficient supporting information; this may be derived from poorly described scientific plausibility and justification of the similarity argument, insufficient evaluation or analysis of the underlying toxicological data or inadequate substance identity profile. In order to meet regulatory expectations, improvement in the definition of the identity profile for both target and source substances as well as compliance with the regulatory needs could be achieved with the distribution of a suitable template to prepare a RAx justification document. One of the important aspects of GRAP is the regulatory scope for the applicability of RAx. In practical terms, this means understanding the standard testing requirements of the framework as well as the specific provisions of how RAx may be applied to adapt those standard requirements.

A general rule for GRAP applicability is the description of each step of the RAx strategy to demonstrate that any possible source substance with correspondent available information has been considered with no bias in the selection or exclusion of source data. The procedure for profiling the target substance to obtain all available source data should be clearly defined to ensure that no relevant study is discarded. After collecting the broadest set of information, data gap-filling should be considered for a well-defined endpoint using a specified procedure. The way to use RAx may depend on the type of information collected, i.e., which scenario selected from the RAAF document is applicable to the specific case (ECHA, 2017b,c). Filtering the available information and the re-organization to group it in order of relevance or mechanistic steps should follow the final aim of the target analysis. This assessment is more complicated if it involves trend behavior that should be quantified in some way. Data collected on possible analogues may lead to discordant results, or poor-quality source data may generate the necessity to deal with outliers and WoE analysis. From the perspective of GRAP, eliminating or downsizing the relevance of a data set is a very sensitive step. It should be handled carefully and in the most transparent way to avoid the generation of increased bias and uncertainty in the final results.

At the end of the procedure, GRAP needs a residual uncertainty assessment with a detailed description of each single step to eliminate any doubt of subjectivity regarding the final outcome and make the final conclusion as reproducible as possible, applying the principle that the same set of data should give the same results independent of the proficiency of the operator.

As already mentioned, good final reporting is essential (Hartung et al., 2019). For this purpose, dedicated templates, e.g., similar to those used by the OECD IATA case studies project (OECD, 2016b), are helpful.

### 3.4 Tailoring to regulators’ needs

Regulatory use of the RAx approach strongly depends on the regulatory framework under which it is used. For that reason, it is also important that the purpose for which the RAx is intended has a clear problem formulation, which could be, for example, classification and labelling, waiving a specific test, or to perform a risk assessment in the scope of a specific regulation. The purpose will also define the level of uncertainty deemed acceptable. For classification and labelling related to a systemic effect, acceptance may depend also on the possible determination of the toxicity category. In risk assessment, less uncertainty also could be acceptable when there is only a small margin between the estimated exposure and risk levels or when sensitive populations such as children, elderly people or pregnant women are exposed (Fig. 8). Acceptance may also depend on whether it is intended to make a negative or positive RAx, in terms of CLP purposes. For a positive RAx, acceptance might be easier when made as a reasonable worst case, whereas for a negative RAx more guarantee should be given that no effects are overlooked. So, the first step is always an in-depth understanding of the regulatory context as well as of the consequences of a wrong decision.

Regardless of the scope, the most important regulatory aspect is the preparation of the RAx justification document, which not only should demonstrate the scientific validity of the approach but also needs to follow the format specified in the relevant regulatory framework.

## 4 Implementation

### 4.1 Capacity building

Capacity building represents a key aspect in the promotion of RAx as a valuable tool for risk assessment. Individuals as well as organizations require the necessary skills to build a satisfactory strategy, decide on new tests and present results in a suitable report. The required field of expertise is wide, covering chemistry, biology, toxicology, computational modelling, experience with the regulatory process and so on, which leads to the conclusion that the knowledge of a single person might not be able to cover all aspects, and thus performing RAx and its assessment adequately requires the activity of a team of experts.

That said, the concept of RAx in its more elaborated form is quite new in toxicology, and to the knowledge of the authors, there is no dedicated course at any university or good textbook available to explain how it works and how to use it.

While there is a growing mass of literature published in peer-reviewed journals and considerable guidance from bodies such as OECD, ECHA, ECETOC, etc., there is no overall knowledge source. Books such as by Cronin et al. (2013) provide a sound basis to the theory, and recent papers such as Patlewicz et al. (2017, 2018, 2019) include updates on the tools that may be applied. Aspects of computational toxicology have been included in under- and postgraduate education for many years, from Bachelor level degrees to PhDs, while RAx as a subject has only recently been included in some academic curricula. RAx will form the core of a new master’s program in computational toxicology^25^. In general, this is not enough to train all aspiring toxicologists.

To make up for the shortfall in education and the desire to train practitioners, a number of courses are on offer. With the exception of regular training on the OECD QSAR Toolbox^16^, most courses are run as one-offs, either as commercial ventures or as continuing education courses at annual toxicology meetings. There is a need to formalize such training capacity to optimize opportunities and increase capacity, for example, by using widespread platforms such as Coursera^26^.

The use of video, e.g., YouTube, as a training means is very helpful to get across key messages. The OECD QSAR Toolbox has for many years provided on-line training in this manner^27^. Other resources are available, e.g., for the US EPA’s Gen-RA tool. One possibility is to bring together all these resources to provide a central place to obtain up-to-date on-line training. This may also show up gaps where more training materials are required.

Many of the computational tools to support RAx typically provide good guidance, and specific training should be organized to improve their use, with the inclusion of examples, recommendations and so on. Users should have the capacity to identify a data gap with a view of how to solve it, whether it is the identification of new source substances, improving TK/TD modelling or performing new tests to confirm a hypothesis. A combination of all available data with proper ranking and quality assessment also requires new expertise.

Notwithstanding that certain data and copyright issues would have to be solved, a collection of real-life case studies to illustrate the key components of RAx would be extremely helpful. Ideally, these would highlight successfully submitted dossiers with a thorough explanation of the key steps and how success was achieved. It is also helpful to demonstrate what caused rejection of RAx cases, illustrating the reasons for non-compliance provided by the relevant authority, as is the case when ECHA does not accept a RAx. Both “best” and “worst” practice examples are instructive and should be shared in the community of RAx performers and assessors for educational purposes.

The preparation of templates for RAx submission can also have a role in guiding new users through a proper application of the RAx approach, in particular if such templates are distributed with examples. Templates could also include the types of reporting formats available from software, e.g., from the OECD QSAR Toolbox^16^, and how these can be adapted and the information content improved. Such templates tend to be useful to store the information from the data retrieval exercise, even though expert knowledge is still required for the argumentation and justification of the RAx.

A key component of successful RAx is its multidisciplinary nature. It is essential that scientists from all relevant disciplines are encouraged to share knowledge of good practice. The organization of inter- and multi-disciplinary workshops can foster communication among users, with possibilities of exchange of information between different fields or at the international level.

Good information exchange requires free access to knowledge hubs, e.g., for tools, guidance, data, templates (Krebs et al., 2019), education, etc., and in this sense, international collaboration should be established to avoid duplication of efforts. Standardization of data-sharing should also improve the mutual acceptability of the methodology and increase confidence in the approach. Other areas suitable for standardization include the terminology used and recommendations for RAx justification. In addition, successful read-across examples should be stored to avoid duplication and to act as a resource. Taking inspiration from the AOP knowledge base, a well-designed web resource and wiki may help with this. In order to improve knowledge and expertise among users, the best opportunity is to improve communication among stakeholders with the creation of databases, either at international or regional levels. This international coordination may also be used to identify gaps and concentrate efforts on the development of new computational tools or experimental methods.

### 4.2 Platforms

It is clear that the availability of specific computational platforms is a prerequisite for a successful application of RAx, aiding in ensuring the objectivity, reproducibility, and transparency of the outcome. That is easy to say, but the applicability is not as clear. The first concern is the quality and homogeneity of source data, keeping in mind the rule “garbage in, garbage out” and, therefore, curation of the available data may represent a key step. Another issue to guarantee that different users arrive at the same conclusion is that algorithms applied in similarity assessment are equivalent. While the demonstration of chemical similarity can be easily automated (Luechtefeld and Hartung, 2017), comparison of biological data is more challenging. In addition to the quality of data sources, there is a total lack of harmonization in reporting the results. While some reporting standards for animal studies exist (Kilkenny et al., 2014), this practice is only on the way for *in vitro* work (OECD, 2017b; Hartung et al., 2019). Analysis of *in vivo* data is different to that of *in vitro* data. There is no standardization of units, ranging from the simple mg or mole/weight expression to the difference in dose among *in vitro*, *in vivo* by gavage or *in vivo* through the feed. A possible solution could be a selection of data, for example considering only GLP studies, but this procedure may trigger other problems, such as the elimination of much important data and the need for detailed rules to accept/disregard data, and therefore introduce another variable that may bring with it errors, bias and/or increased uncertainty. Weighting of source data is essential and may represent a possible solution if performed in a transparent way. Principles and key elements for establishing a WoE for chemical assessment are contained in an OECD guidance document (OECD, 2019) following ECHA, which published a template for reporting WoE^28^, but guidance on how to combine different lines of evidence, how to best assign differential weights, and how to quantitatively combine those lines with the aim of minimizing residual uncertainty as well as how to manage discordant results is still missing.

Other important hurdles lie in the legal aspects of the right to refer to proprietary data as is the case with the majority of study reports. Even the public ECHA database has restrictions in the right to use, although a note explicitly allows download and use “*to improve the safe use of chemicals, enable innovation and help avoid the unnecessary testing of chemicals on animals*”^29^.

At the moment, there are two main available platforms: the OECD QSAR Toolbox and the EPA CompTox Chemicals Dashboard. The OECD QSAR Toolbox^16^ is based on a variety of publicly available databases for mammalian and environmental effects and includes the ECHA public database of registered substances. It is structured in a mechanistic way that enables the reconstruction of the grouping process relevant for each endpoint. The EPA CompTox Chemicals Dashboard^18^ is compiled from sources including the EPA’s computational toxicology research databases and other US public domain databases. The GenRA approach, which was developed with the aim of systematically and objectively evaluating the performance and quantifying the uncertainties associated with read-across predictions, is included (Shah et al., 2016; Williams et al., 2017; Helman et al., 2018). The ideal future solution should exploit both platforms, and this is what the private tool REACHacross is proposing^30^ (Luechtefeld et al., 2018b) through an advanced machine learning algorithm for identification of the most relevant chemical analogues ensuring reliable results, claiming to be just as accurate as other available approaches and animal testing (Luechtefeld et al., 2018a,b).

There are also many other private tools that can be useful. Patlewicz et al. (2017) performed a review of available *in silico* tools for grouping and *inter alia* compared them with a RAx workflow. The recent ICCVAM RAx working group publication also provides a description of freely available tools and insight into those used by regulatory agencies to support decision-making (Patlewicz et al., 2019). A fundamental prerequisite of any tool is the clear definition of the approach, with defined integration of QSAR assessment and the possibility to backtrack data history and version of the tools, as well as a simple format output adhering to the OECD principles for QSAR validation (OECD, 2007).

A very interesting initiative is the VegaHub^31^ that developed specific tools, such as ToxRead, a software that builds up graphs connecting the target chemical and the structural alerts with the most similar compounds, to be used in combination with ToxWeight, which provides a WoE assessment joining QSAR and RAx, and ToxDelta focused on the molecular structure differences, namely the dissimilarity features (Gini et al., 2014; Golbamaki et al., 2017).

Another available platform and public tool is AMBIT2^32^, which is an open chemoinformatic system designed to support companies by facilitating chemical safety assessment. The AMBIT2 system consists of a database including more than 450,000 chemical structures, the REACH dataset of 14,570 substances, as well as the EFSA OpenFoodTox database. It contributes to the safer use of chemicals and a reduction in testing and innovation cost by making it easier for companies to comply with regulations governing chemicals. Several *in silico* prediction models (e.g., Toxtree, Vega) are integrated in AMBIT2. The import and export functions are enhanced in AMBIT2, which allows communication with a variety of additional prediction models such as knowledge-based expert systems for toxicity and metabolism.

The use of several computational tools (both commercial and free) can support the different phases of the RAx workflow, e.g.:

- tools/databases to search for potential analogues – by structural similarity, experimental data availability for the target-endpoint, combined search (structural similarity & target endpoint data availability) or mechanistic similarity;
- tools to assess similarity between target and source compounds through structural similarity, mechanistic similarity, physicochemical and ADME similarity;
- tools to predict potential metabolism and metabolic products;
- QSAR predictors to predict target endpoint-related properties.

The big advantage of automation of source substance selection lies in the evaluation of many substances in a limited time. These approaches need to be used with care. They often simply identify “similar” analogues on the basis of chemical fingerprints, which may work for some endpoints but leaves the problems of activity cliffs if the fingerprints are not appropriate (Mellor et al., 2019). An example from skin sensitization is that a small change in structure can change a non-sensitizing molecule to one that can cause sensitization and *vice versa*. Still, already relatively simple nearest neighbor approaches were reported as quite successful in predicting this hazard (Luechtefeld et al., 2016b).

The addition of specific modules may make a difference from the perspective of regulatory acceptability. It is now clear that TK and metabolism are essential. When no data are available from *in vivo* studies, new models should be developed with the support of specific *in vitro* experiments. Given the complexity of RAx, automation only seems to make sense with respect to analogue selection, but for regulatory acceptance also a comparison of other physicochemical and toxicological properties is required.

The extent of familiarity with and uptake of automated tools as well as resources and guidance is mixed across the different agencies. In some cases, there is a bias towards using QSAR approaches vs RAx, and the tools used vary between using the OECD Toolbox or commissioning the development of new tools relying on other data streams. Harmonization and standardization of the approach as well as documenting the use of QSAR models as part of a workflow is desirable (Myatt et al., 2018).

### 4.3 Non-regulatory RAx

RAx may also be useful outside the regulatory scope since it represents a valuable tool for research and early prediction. Developers of RAx systems should consider applicability in other scientific areas and possible users such as chemists, toxicologists or in education, involving teachers and students.

RAx can find application in research and development contexts, e.g., at screening and prioritization level for the identification of hazardous compounds to drive experimental testing or even to drive the synthesis of new molecules (“benign by design” concept). These areas are also known as green toxicology, which is a sector of the green chemistry movement (Maertens et al., 2014; Maertens and Hartung, 2018) asking for less toxic products, safer processes, and less waste and exposure. In these contexts, grouping approaches should be more promising and useful than analogue approaches, with possible identification of trends that can be very useful in the selection of a new lead. Automated RAx tools can enhance the identification of unusual combinations of chemicals with the evaluation of the impact on toxicity generated by small changes in the chemical structure. (NAM-based) RAx could also support green chemistry in the selection of substances suitable for replacing substances of very high concern (SVHC).

Specific use of RAx in academia can go beyond pure research as is the case for industry R&D. RAx should be part of teaching courses in toxicology and other disciplines, with practical sessions on case studies and emphasis on the importance of RAx as a necessary professional qualification. RAx should be a valuable tool in mechanistic understanding as an ideal complement of pure computer modelling systems. Academia should reason in a reverse way, i.e., frame their research questions and present their data in a machine-readable way with reports that contain all useful information to enrich databases for RAx exploitation. Last but not least, the development and optimization of RAx tools represents a thriving research field for academic teams.

There are emerging applications also inside regulatory institutions. For example, two EPA Research Centers are engaged in the area of RAx. Within the National Center for Environmental Assessment (NCEA), RAx is routinely applied as part of the Superfund program for provisional peer-reviewed toxicity values (PPRTVs). The workflow is documented in Wang et al. (2012). Beyond federal research centers, there are a number of opportunities under development to implement RAx in green chemistry approaches and screen chemicals during research and development by industry, e.g., the Leadscope models^33^ and the associated *in silico* protocols effort.

## 5 Conclusion / recommendations

RAx is potentially a major tool for risk assessment. The power of RAx lies in the possibility of combining data in a global analysis, including:

- Data on analogues,
- Data on grouping, with identification of trends across a group,
- QSAR model predictions,
- *In vitro* tests, with a focus on AOPs,
- TK/TD analysis.

This approach in its more elaborate form is relatively new and requires specific expertise that at the moment is still limited. The roadmap for implementation and confident use of RAx requires actions in many areas with the collaboration of different stakeholders.

A very important step is the generation of a template for RAx submission to the authorities with specific instructions on GRAP. In order to increase the confidence in the tool, the creation of examples that would be considered acceptable by regulators is key. Examples should include cases with compounds with high, low or no activity. A detailed analysis of uncertainty is a very important part of this. To this aim, workshops and symposia may contribute in the dissemination of the RAx principles.

Simultaneously, other actions should support raising awareness of regulators inside national authorities that RAx is a valid alternative, with the potential to increase confidence in the final outcome as it is based on a reasoned approach with a scientific justification of the mechanism. For this purpose, retrospective examples comparing both the traditional approach and the assessment performed with RAx should be prepared and presented.

On the other side, risk assessors from industry should also learn how to properly apply RAx. There is still much ignorance and apprehension about using RAx in industry, and whilst much guidance and templates for reporting are available, real examples of successful, i.e., accepted, RAx for data gap-filling would be beneficial. The industry needs to be reassured that the effort in preparing a rigorous RAx justification is largely repaid by regulatory acceptance without the need for additional *in vivo* studies.

Better harmonization of legislation is desirable. That should happen internationally, as it is unacceptable that a good RAx would be, for example, accepted in the EU and rejected in Japan or *vice versa*. It is not surprising that industry is reluctant to invest money into a strategy that is not accepted in other parts of the world. Unfortunately, the problem is not limited to crossing national borders, as sometimes important differences are present among different regulations enforced in the same region. Political action towards harmonization is necessary.

Another important aspect is education. RAx should be part of the program in university courses in toxicology and other related subjects, in order to provide another useful tool for research and implement NAM approaches.

RAx approaches are a dynamic and continually evolving development. The area of mechanistic elucidation of toxicity pathways along with the increase of available information and computational tools is expanding dramatically, and each assessment should be updated in parallel with the commitment to incorporate new evidence into existing RAx packages. These technologies offer the possibility of refinement of RAx justifications, which may lead to greater openness towards acceptance on the regulators’ side. International organizations such as the OECD have a leading role to play in supporting the process definition.

The fourth sector that is affected by RAx is contract research organizations (CROs) that usually perform new tests on behalf of industry and in many cases provide advice on test strategies. CROs should be aware of RAx possibilities and should implement NAMs in the list of their services. Widespread implementation will facilitate a decrease in the cost of *in vitro* tests, which is desirable to encourage their application to support RAx justification.

In addition, due to a general lack of strict guidance/rules for GRAP, chances of regulatory failure of a RAx submission are considerably higher compared to, e.g., submitting the “traditional” standard information dataset as required by REACH. Against this background, many companies will prefer higher initial spending with guaranteed regulatory acceptance over an initially smaller investment, which might result in overall much higher costs when a RAx submission is rejected.

Last but not least, there is a need for the development of new tools for building RAx. This part is more complex. First of all, there should be an international agreement for data-sharing and free access to high-quality reports. Then there is the need for new databases with mechanistic information, combined with TK/TD data. In fact, at the moment, the request is to apply RAx simply to waive an extensive *in vivo* test, but considering the limitations of *in vivo* tests, the future trend is to develop a tool that increases predictivity and reliability by combining RAx approaches with multiple NAMs.

## Figures and Tables

**Tab. 1:** Some examples of available public case studies on the applicability of RAx for regulatory purposes, as described in the text

Projects	Description	Main documents
**ECHA - COLLA project (2017–2018)**	The project aimed to improve the information used to decide on the needs for further regulatory risk management with the involvement of Member State competent authorities and concerned registrants. Two different elements were tested: addressing substances by groups and early interaction with registrants. The project demonstrated an increase in effectiveness, providing a better picture of the gaps and the points of concern.	ECHA, 2018
**SEURAT-1 (2009–2014)**	The primary goal of the read-across case study within the project was to increase confidence in the read-across assessment by using data from alternative methods. The CS were run under 2 conditions: read-across without additional new approach data; addition of NAM data (primarily from ToxCast and from application of the alternative methods developed within the initiative).	Berggren et al., 2015; Schultz et al., 2015, 2017a,b; Mellor et al., 2017
**EU-ToxRisk project (2016–2021)**	In the EU-ToxRisk project, the Read-Across Case Study Strategy includes assessment of toxicokinetics, both in the *in vitro* experimental set up and the extrapolation to safe human doses. These are integrated into the design of EU-ToxRisk case studies through the use of ADME models and generation of ADME *in vitro* data. The results of the NAM-supported read-across case studies are compiled and sent for evaluation to regulators as a “mock submission”. The experience and learnings gained during the project will be compiled in a NAM-based read-across guidance.	Escher et al., 2019; Rovida et al., in preparation
**OECD Cooperative Chemicals Assessment Programme (CoCAP); IATA Case Studies Project (2015-current)**	The objective of the IATA Case Studies Project is to increase experience with the use of IATA by developing case studies, which constitute examples of predictions that are fit for regulatory use. The aim is to create a common understanding of using novel methodologies and the generation of considerations/guidance stemming from these case studies. Case studies submitted by OECD member countries are reviewed regularly. Learnings and lessons derived from such reviews are regularly published as OECD reports.	OECD, 2018

**Tab. 2:** Questions to assist in the identification of potential sources of uncertainty that may impair RAx prediction (Schultz et al., 2019)

**1 The context of, and relevance to, the regulatory use of the read-across prediction as defined by appropriate problem formulation**	– Is the regulatory purpose of the read-across prediction clearly defined? – Is the acceptable level or degree of uncertainty for the stated purpose defined? – Is the stated acceptable level or degree of uncertainty appropriate for the stated regulatory purpose?
**2 Type of category/group including the definition of the applicability domain**	– Is the read-across approach (e.g., analogue or category) clearly reported? – Are the target and source chemicals clearly identified? – Is the applicability domain of the analogue or category defined? – Do target and source chemicals fit within the defined applicability domain?
**3 The premise or hypothesis of the read-across**	– Is the hypothesis on which the read-across is based clearly stated and presented in sufficient detail to be assessed?
**4 Mechanistic plausibility including completeness of the understanding of the MoA or AOP**	– How clearly does the hypothesis state the chemical and biological mechanisms underpinning the toxic effect being read across? – Is there sufficient experimental information provided to support the proposed chemical and toxicological mechanisms? – How extensively does the experimental information provided support the mechanistic plausibility and/or the AOP or MoA on which the read-across is based?
**5 Similarity in chemistry**	– Are the chemical structures (i.e., 2D structure, isomers, SMILES and molecular formula) reported for the derivatives used in the read-across? – Are the dissimilarities in chemical structure reported, and are they toxicologically relevant? – Are the relevant molecular and physicochemical properties (e.g., molecular size, hydrophobicity, solubility, volatility, degradation, etc.) reported for the derivatives used in the read-across? – Are the dissimilarities in molecular and physicochemical properties reported, and are they toxicologically (or pharmacokinetically) relevant?
**6 Toxicodynamic similarity**	– Is there sufficient and consistent toxicodynamic information provided to establish similarity in the hazard of the derivatives used in the read-across?
**7 Toxicokinetic similarity**	– Is there sufficient ADME information provided to establish toxicokinetic similarity for the derivatives used in the read-across? – Are any dissimilarities in ADME properties (and, as appropriate, metabolism/degradation) toxicologically relevant?
**8 The quality of the apical endpoint data used to fill the data gap**	– Is the performance (e.g., reliability, accuracy, precision, repeatability and reproducibility) of the data read across reported clearly? – Has the quality of the data to be read across been assessed, and are they sufficient to meet the purpose of the exercise, i.e., complete and of sufficient quality?
**9 The consistency in the effects and severity of the apical *in vivo* hazard and their concordance with regards to the intermediate and apical effects and potency data**	– Is the qualitative expression of the data reported, and is it consistent among the source chemicals? – Is the potency of the hazard reported, and is it consistent among the source chemicals? – What are the temporal relationships between relevant endpoints? – What are the dose-response relationships between relevant endpoints?
**10 Strength or robustness of the supporting data sets**	– How extensively are the relevant or key events either empirically measured and/or modelled by appropriate *in silico, in chemico* and *in vitro data?* – Is the performance (e.g., reliability, accuracy, precision, repeatability and reproducibility) of the supporting methods adequately reported?
**11 The weight-of-evidence (WoE) supporting the prediction**	– Is there consistency in the supportive information (e.g., structural alerts) between analogues or within the category? – How many and how large are the dissimilarities in the supporting information (i.e., data gaps)?
**12 Documentation and written evidence provided**	– Is the read-across prediction adequately documented? – Does the evidence support the hypothesis that the uncertainty is acceptable for the stated purpose (as per Question 1)?

## Footnotes

1
https://newsletter.echa.europa.eu/home/-/newsletter/entry/reach-compliance-an-agency-priority-for-2019

2
http://caat.jhsph.edu/programs/workshops/readacross.html

3
https://www.epa.gov/reviewing-new-chemicals-under-toxic-substances-control-act-tsca/chemical-categories-used-review-new

4
https://www.epa.gov/sites/production/files/2015-05/documents/09.pdf

5
https://www.epa.gov/tsca-screening-tools/chemical-assessment-clustering-engine-chemace

6
https://ntp.niehs.nih.gov/pubhealth/evalatm/3rs-meetings/past-meetings/commprac-2016/commprac-2016.html

7
https://www.canada.ca/en/health-canada/services/chemical-substances/substance-groupings-initiative.html

8
https://www.canada.ca/en/health-canada/services/chemical-substances/chemicals-management-plan/science-committee/meeting-records-reports/committee-report-november-4-5-2014.html

9
https://www.canada.ca/en/health-canada/services/chemical-substances/fact-sheets/application-weight-of-evidence-precaution-risk-assessments.html

10
https://www.nicnas.gov.au/New-scheme-1-July-2020/Key-information-about-the-new-scheme#draftgeneral

11
www.nite.go.jp/en/chem/qsar/hess-e.html

12
www.concawe.eu/cat-app/

13
https://www.fda.gov/newsevents/newsroom/pressannouncements/ucm595622.htm

14
https://www.h2020gracious.eu

15
https://www.gmp-compliance.org/gmp-news/emas-reflection-paper-on-the-qualification-of-non-genotoxic-impurities

16
https://www.oecd.org/chemicalsafety/risk-assessment/oecd-qsar-toolbox.htm

17
http://dissemination.echa.europa.eu/Biocides/ActiveSubstances/1232-01/1232-01_Assessment_Report.pdf

18
https://comptox.epa.gov/dashboard

19
http://qsar.food.dtu.dk

20
http://www.oecd.org/chemicalsafety/risk-assessment/iata-integrated-approaches-to-testing-and-assessment.htm

21
https://aopwiki.org/aops

22
https://www.eu-toxrisk.eu/media/articles/files/EU-ToxRisk_Press%20Release_FINAL.pdf

23
https://www.concawe.eu/wp-content/uploads/2017/01/cr231-petroleum_products_and_reach-2014-01756-01-e.pdf

24
https://ec.europa.eu/jrc/en/eurl/ecvam/alternative-methods-toxicity-testing/validation

25
https://www.ljmu.ac.uk/study/courses/postgraduates/computational-toxicology-msc

26
https://www.coursera.org/

27
https://qsartoolbox.org/support/

28
https://echa.europa.eu/documents/10162/17169198/wo_eu_uncertainty_background_en.docx/4f2b49ab-ade0-6ee3-e977-8abe00c21c23

29
https://echa.europa.eu/-/data-on-15-000-chemicals-now-available-to-use

30 www.ulreachacross.com, the website has been discontinued and an advanced software been made available as UL Cheminformatics Tool Kit

31
www.vegahub.eu/portfolio-item/toxdelta/

32
http://cefic-lri.org/toolbox/ambit/

33
http://www.leadscope.com/
